# A realistic two-strain model for MERS-CoV infection uncovers the high risk for epidemic propagation

**DOI:** 10.1371/journal.pntd.0008065

**Published:** 2020-02-14

**Authors:** Tridip Sardar, Indrajit Ghosh, Xavier Rodó, Joydev Chattopadhyay

**Affiliations:** 1 Department of Mathematics, Dinabandhu Andrews College, Kolkata, India; 2 Agricultural and Ecological Research Unit, Indian Statistical Institute, Kolkata, India; 3 ICREA &CLIMA (Climate and Health Program), ISGlobal, Barcelona Institute for Global Health, Barcelona, Spain; Institute for Disease Modeling, UNITED STATES

## Abstract

Middle East Respiratory Syndrome Coronavirus (MERS-CoV) causes severe acute respiratory illness with a case fatality rate (CFR) of 35,5%. The highest number of MERS-CoV cases are from Saudi-Arabia, the major worldwide hotspot for this disease. In the absence of neither effective treatment nor a ready-to-use vaccine and with yet an incomplete understanding of its epidemiological cycle, prevention and containment measures can be derived from mathematical models of disease epidemiology. We constructed 2-strain models to predict past outbreaks in the interval 2012–2016 and derive key epidemiological information for Macca, Madina and Riyadh. We approached variability in infection through three different disease incidence functions capturing social behavior in response to an epidemic (e.g. Bilinear, BL; Non-monotone, NM; and Saturated, SAT models). The best model combination successfully anticipated the total number of MERS-CoV clinical cases for the 2015–2016 season and accurately predicted both the number of cases at the peak of seasonal incidence and the overall shape of the epidemic cycle. The evolution in the basic reproduction number (*R*_*0*_) warns that MERS-CoV may easily take an epidemic form. The best model correctly captures this feature, indicating a high epidemic risk (1≤*R*_*0*_≤2,5) in Riyadh and Macca and confirming the alleged co-circulation of more than one strain. Accurate predictions of the future MERS-CoV peak week, as well as the number of cases at the peak are now possible. These results indicate public health agencies should be aware that measures for strict containment are urgently needed before new epidemics take off in the region.

## Author summary

There is currently no way to anticipate MERS-CoV epidemic outbreaks and strategies for disease prediction and containment are largely undermined by the limited knowledge of its epidemiological cycle. Not an effective treatment nor a vaccine for MERS-CoV exist to date. Instead, using three two-strain mathematical models that incorporate human social behavior as different disease incidence functions (e.g. bilinear, non-monotone and saturated), the best model combinations successfully anticipate the occurrence of the peak week in the season and the incidence at the peak. Our results confirm there are currently 2 strains co-circulating in the most populated regions in Saudi Arabia and highlight the high risk for large epidemic outbreaks, while the role of super-spreaders appears irrelevant for disease spread.

## Introduction

**Fig 1:**
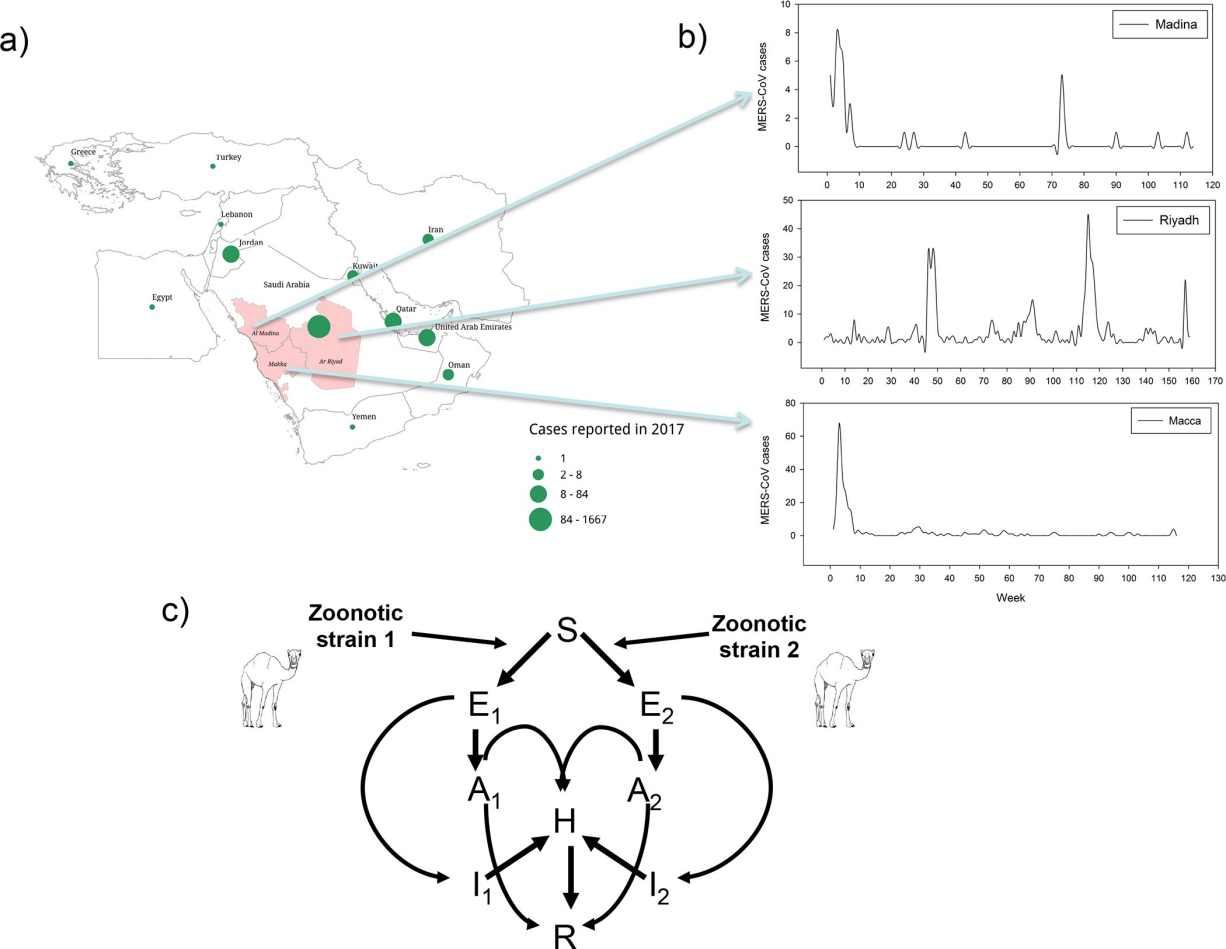
Distribution of MERS-CoV weekly cases in Riyadh, Macca and Madina provinces during July, 2013—June, 2016. (a) The geographic distribution of MERS-CoV cases reported in 2017 in the Middle East. (b) Time series of weekly incidence data of MERS-CoV in three major provinces, Riyadh, Maccaand Madina, respectively. (c) Two strain Models flow diagram considering asymptomatic, symptomatic, hospitalized, and Zoonotic transmission. Open source KML map for the Middle East was kindly obtained and redrawn by Josep-BoyardMicheau from https://community.qlik.com/t5/Qlik-Sense-Enterprise-Documents/GCC-Middle-East-country-boundary-KML-maps-KML-Shapefile/ta-p/1478595.

The first case of Middle East Respiratory Syndrome Coronavirus (MERS-CoV) infection was identified in Saudi Arabia in 2012[1–5]. Since then the country suffers from repeated outbreaks of MERS-CoV in different provinces (Fig 1A and 1B)[2]. There are two main routes of MERS-CoV transmission: animal-to-human and human-to-human[4–6]. It is suspected that dromedary camels are the source of human infections[1] but the transmission route of MERS-CoV to humans is yet not well understood (Fig 1C). However, transmission from camels to humans is confirmed from the isolation of near-identical strains of MERS-CoV from epidemiologically coexisting camels and humans[7]. The patients might be exposed to MERS-CoV by consumption of raw camel products[8] (e.g. milk and dairy products, raw meat, etc.). Meanwhile, human-to-human transmission has been reported in society and hospital settings[9–36] with the virus being transmitted between humans during close human-to-human contact through droplets of respiratory secretions. Potential propagation to nearby and more distant regions is also a high-risk possibility as an outbreak of MERS-CoV is likely to emerge in areas such as nearby countries in the Middle East and eastern Africa where the camel trade connects the different regions (Fig 1). Unfortunately, as for now, MERS-CoV vaccines are only at the preclinical phase[10], increasing our understanding of its epidemic potential and knowledge on the drivers of MERS-CoV variability might help to achieve better preparedness ahead of forthcoming epidemics.

The ability to predict disease outbreaks will provide a mechanism for policymakers and health-care services to respond to epidemics in a timely manner, reducing the impact and maximizing the limited resources available to be deployed[11]. The timing and severity of infectious disease outbreaks, two matters of considerable public-health relevance, are the main challenges when attempting to predict disease outbreaks[11–14]. Attempts to set up prediction frameworks for anticipating epidemics for other diseases such as dengue and influenza were pursued in the recent past with different degree of success, but clear added value[11–16]. These systems proved effective to better anticipate future outbreaks and increase our understanding on mechanisms driving disease variability. To this end, a threshold quantity that is most important to anticipate the risk of future outbreaks is the basic reproduction number (*R*_*0*_). There are quite a few studies that estimated *R*_*0*_ for the current MERS-CoV outbreak. Majumdar et al.[28] for instance, estimated *R*_*0*_ for Jeddah and Riyadh using a function called Incidence Decay with Exponential Adjustments (IDEA). They found that the estimate of *R*_*0*_ in Jeddah and Riyadh are in the ranges (3.5–6.7) and (2.0–2.8), respectively. A stochastic epidemiological model of MERS-CoV with zoonotic and human-to-human transmission was considered by Poletto et al.[29] to quantify the rates of generation of cases from those two transmission routes. They found that spring 2014 cases led to the increase in transmission rates, which brings *R*_*0*_ to values above unity. Heishet al.[32] used a simple mathematical model to trace the temporal course of South Korea MERS-CoV outbreak. They estimated *R*_*0*_ to be in the range of 7.0 to 19.3. Instead, Nishiura *et* al.[30] estimated the expected number of secondary cases following the importation of an index case (countries other than KSA, Qatar and UAE), using a branching process type modelling approach. They also suggested that even if *R*_*0*_ is below unity, a large cluster of cases with multiple generations might occur. The aforementioned studies indicated that the basic reproduction number is above unity, although there are few studies that suggest *R*_*0*_ is less than one[4,17,18]. However, at the same time, intriguing differences in observed and predicted scenarios clearly suggested other important factors might be missing in previous models built for these MERS-CoV case studies. For instance, in a recent survey for Mers-CoV in over 1300 Saudi Arabian camels, Sabiret al.[1] found that camels share three coronaviruses (CoV) species with humans. Among them, one has been dominant in the region since December 2014 and led to the human MERS-CoV outbreaks occurring in 2015. The wide species range of CoVs and their propensity to cross species boundaries suggest that more MERS-CoV uncontained outbreaks may likely occur in the future. Zumlaet al.[19] also suggested that MERS-CoV species can mutate to have increased inter-human transmissibility. Cottenet al.[37] used MERS data from the period May to September 2013. They found four Saudi Arabia MERS-CoV phylogenetic clades, with 3 clades apparently no longer contributing to current cases, therefore not appearing in the saliva of camels. They also show that the ancestors of most of the viral clades originated in Riyadh (See Cotten et al.[37]). Recently, strain variability in MERS-CoV infection was confirmed in 51 respiratory samples from 32 patients, confirming that more than one strain of human MERS-CoVis present[20]. The fact that more than one MERS-CoV strain is currently circulating in Saudi Arabia should be properly accounted for as this presence may have substantially contributed to amplify the transmission intensity producing repeated MERS-CoV changes in incidence through their periodic reintroduction into human populations[19]. Furthermore, Chan et al.[34] characterized MERS-CoV viruses from dromedaries in Saudi Arabia and Egypt and compared them with a human MERS-CoV reference strain. They isolated three dromedary strains, two from Saudi Arabia and one from Egypt. The human and dromedary MERS-CoV strains had similar viral replication competence in Vero-E6 cells and respiratory tropism in ex-vivo cultures of the human respiratory tract. They also suggested that dromedary viruses from Saudi Arabia and Egypt are probably infectious to human beings[34].

## Methods

### a) Disease incidence functions

**Fig 2:**
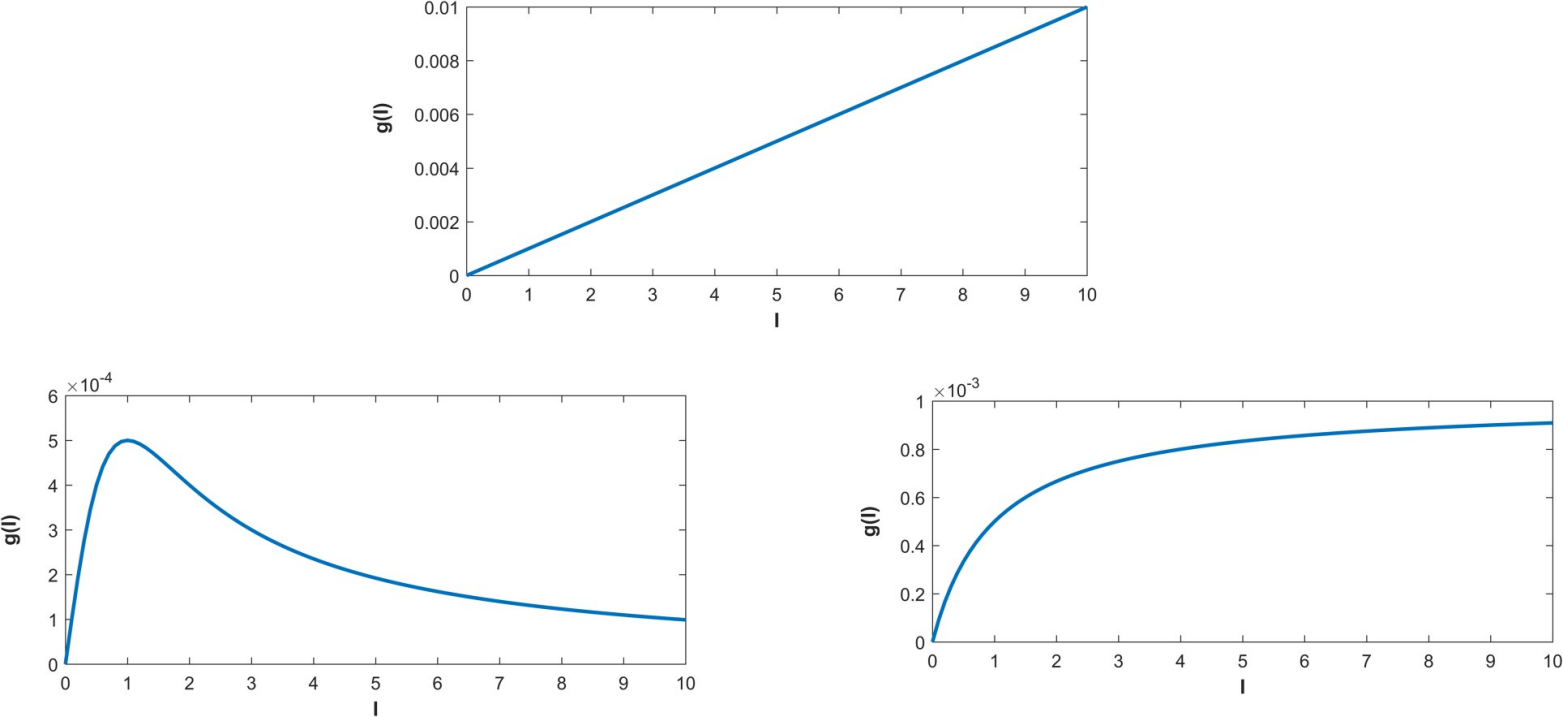
Representation of the three incidence functions, g(I). Top: g(I) = βI, bilinear incidence function. Bottom-left: g(I) = $\frac{\beta I}{N(1+\alpha I^{2})}$, non-monotone incidence function and; bottom-right: g(I) = $\frac{\beta I}{N(1+\alpha I)}$, saturated incidence function. Here, N = 1000, α = 1 and β = 1.

We constructed three different 2-strain models for MERS-CoV that consider community human-human, hospital human-human, and passive zoonotic transmission (Fig 1C and see also S1–S18 Tables). Models derived also incorporate the effect of strain variability with strain-1 as the dominant strain (Fig 1C and S1–S18 Tables). Variability among models is based upon three different disease incidence functions (e.g. BL, NM and SAT models [21–23] and see formulation in Table 1A and Fig 2).

**Table 1:** (A)Description of the incidence functions for the three models considered. (B)Main parameters of the three 2-strain models.

A					
Incidence functions		Description		Source	
$f(I)=\frac{\beta_{1}SI}{N}$		Bilinear incidence function		23	
$f(I)=\frac{\beta_{1}SI}{N(1+\alpha I^{2})}$		Non-monotone incidence function		21, 22	
$f(I)=\frac{\beta_{1}SI}{N(1+\alpha I)}$		Saturated incidence function		21	
B					
Symbol	Description		Range/Fixed		Sources
*μ*	Natural birth/death rate of human		2.5924×10^−4^Week^-1^		24
*β*_1_	Transmission coefficients of symptomatic and asymptomatic cases		*[0*, *100]* Week^-1^		Estimated
*θ*	Measure of variability of two strains		*[0,1]*		Estimated
*ρ*	Reduction in transmission due to less infectiousness of asymptomatic infected individuals		*[0,1]*		Estimated
*β*_2_	Transmission rate from hospitalized to susceptible individuals		*[0*, *100]* Week^-1^		Estimated
*β*_3_	Import rate of external zoonotic infection		*[0, 10]* Week^-1^		Estimated
*p*_1_	Proportion of susceptible that get infected from hospitalized individuals with strain-1		[0,1]		Estimated
*p*_2_	Proportion of susceptible that get infected from zoonotic infection with strain-1		[0,1]		Estimated
*p*_3_	Proportion of exposed individuals that become symptomatic infected		0.553		4
*c*_1_	Undetected entry rate of strain-1 asymptomatic class to hospitalized class		[0,5]		Estimated
*c*_2_	Undetected entry rate of strain-2 asymptomatic class to hospitalized class		[0,5]		Estimated
$\frac{1}{\sigma }$	Incubation period		0.7429 Week		19,25,26
*K*	Recovery rate of asymptomatic individuals		1.4 Week^-1^		27
*λ*	Hospitalization rate of symptomatic individuals		4.4824 Week^-1^		4
*γ*	Recovery rate of hospitalized individuals		1 Week^-1^		4
*δ*_2_	Disease induced death rate of hospitalized individuals		Data		2
*α*_1_	Measure of inhibitory effect of strain-1 (only for non-monotone and saturated incidence models)		[0,500]		Estimated
*α*_2_	Measure of inhibitory effect of strain-2 (only for non-monotone and saturated incidence models)		[0,500]		Estimated

The top panel in Fig 2 represents the BL force of infection. Here, as the number of infected individuals increases the disease transmission also increases linearly. The bottom-left panel represents the NM incidence function. Biologically this incidence function can account for “psychological effects” [38–39]. In the presence of psychological effects, initially the disease transmission rate increases rapidly when the number of infected individuals is small. However, this rate falls also rapidly in the presence of a large number of infected persons in the community. As in the case of MERS-CoV infections the CFR is about 40%, psychological effects for this infection represents the effect in the community of fear of becoming infected. This fear effect reduces the transmission rate rapidly in the presence of a high number of infections in the community. The bottom-right panel of Fig 2 represents the SAT incidence function. Biologically this incidence function represents the crowding effect in disease transmission. This crowding effect explains how the number of new infections becomes constant as the number of infected individual increases. This effect is known to reproduce the awareness effect of the disease during the course of an epidemic.

### b) Super-spreaders and 1-strain vs 2-strain models

**Fig 3:**
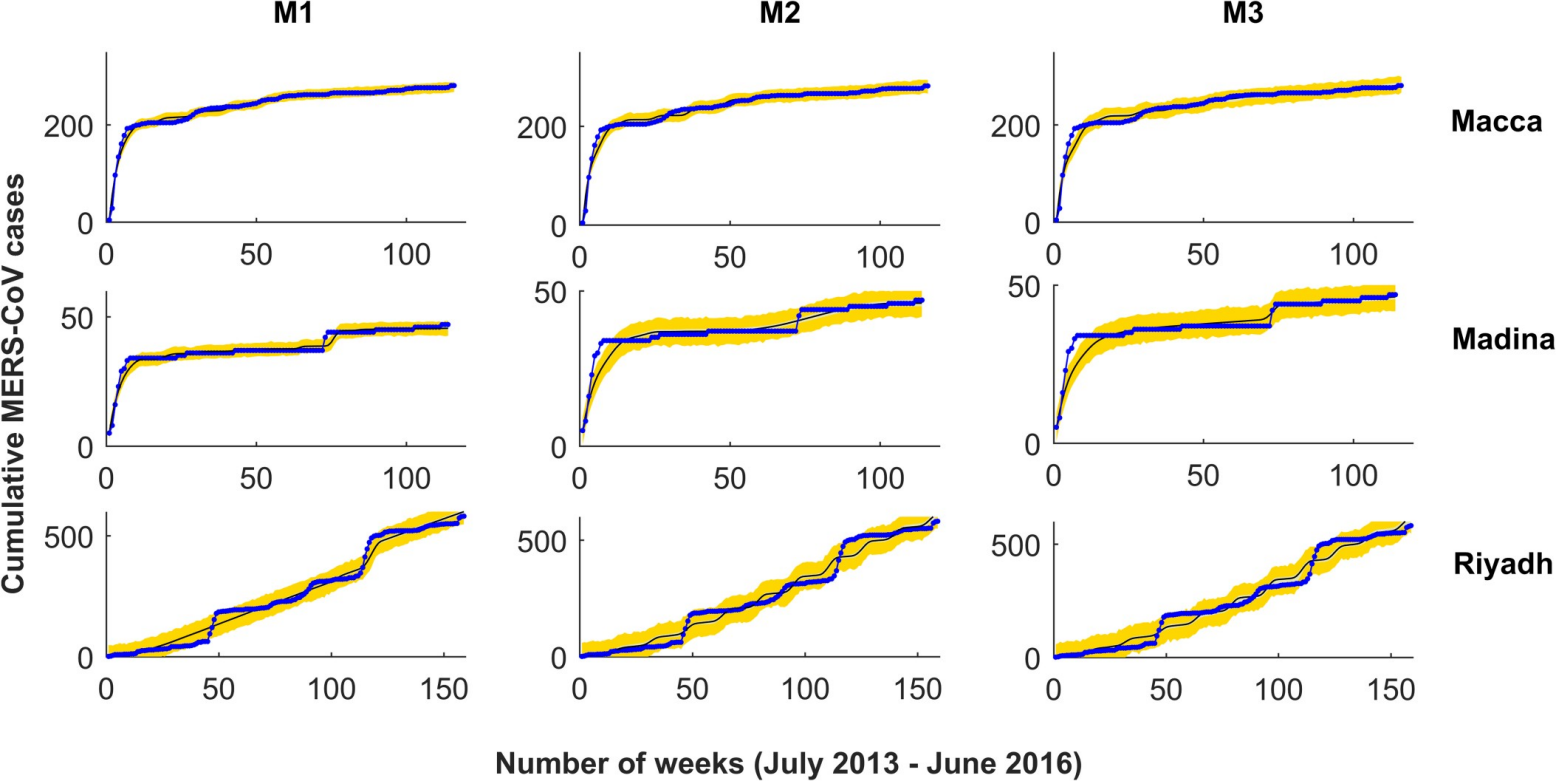
Model simulations fitted to accumulated MERS-CoV clinical cases in Macca, Madina and Riyadh. Observed data points are shown in blue and the solid line depicts the model solutions. The three two-strain models fitted to cumulative MERS-CoV cases are: M1: 2-strain model with bilinear incidence, M2: 2-strain model with non-monotone incidence, and M3: 2-strain model with saturated incidence.

**Fig 4:**
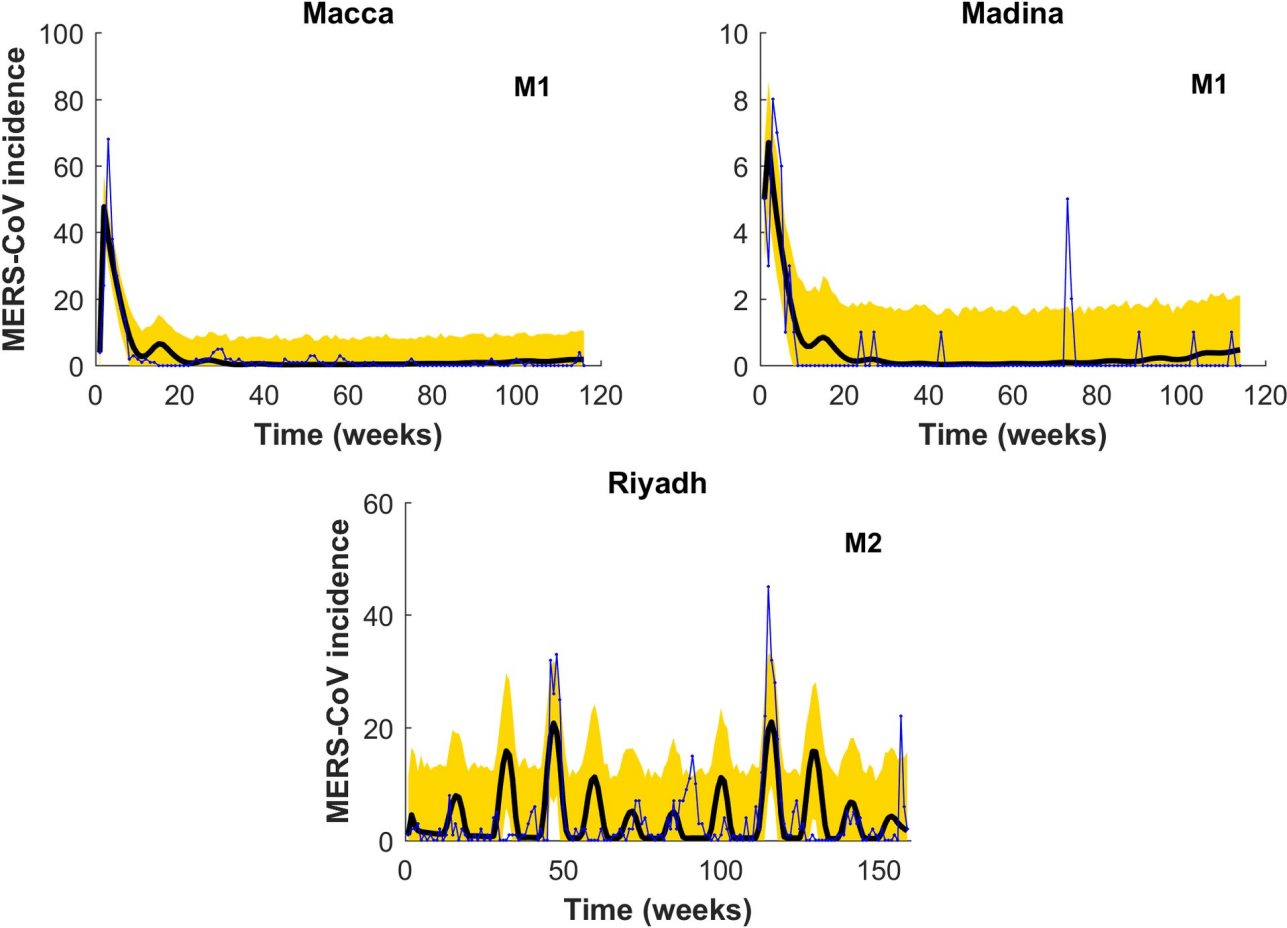
Same as for Fig 3 but predictions showing the best model for MERS-CoV incidence in each of the three regions. Notice that whereas M1 provides the best prediction for both Macca and Madina (top), M2 instead does it for the two-strain situation occurring in Riyadh. Blue line is MERS-CoV cases, solid black line predictions and yellow area denotes 95% confidence interval for predictions.

Furthermore, we investigated the potential effects of super-spreading events by incorporating an additional compartment for super-spreaders to our 2-strain model. We also consider the effect of variable zoonotic transmission by incorporating dynamics of the camel population into our 2-strain model. Information about the calculation of the epidemiological parameter values for the newly proposed 2-strain models is provided in Table 1B. Data used corresponds to weekly cases of MERS-CoV for the three provinces in Saudi Arabia where most clinical cases for MERS-CoV occurred for the time intervals July, 6^th^ 2013 till June, 28^th^ 2016 (Riyadh), April, 7^th^2014 till June, 26^th^2016 (Macca), and April, 20^th^2014 till June, 26^th^2016 (Madina), respectively (Fig 1A). Main parameters of the different models are provided in Table 1B. Parameters estimates are obtained by fitting the models to new MERS-CoV hospitalized weekly data[40]. We also estimated from data itself some unknown initial conditions for the model. Delayed Rejection Adaptive Metropolis algorithm is used here to sample the 95% confidence.The goodness of fitsto compare among 2-strain and single strain models were tested by determining the respective AIC and BIC values (See S19 Table). To further compare among predictive capabilities of these models with regard to those of their single-strain versions, predictions of raw clinical cases were attempted. Albeit some discrepancies exist, fairly accurate results were obtained despite the low numbers and highly stochastic nature of the three datasets and the nearly operational capacity of these models (Fig 3). In fact, near-operational systems usually require a much longer training period than the scarce three years employed here. Differences arising from the comparison of simulated and observed cases may also be due to the fact that we are fitting the 2-strain and single strain models to cumulative MERS-CoV cases, rather than to new cases.^.^The procedure for model adjustment is as follows: for approaching near-operational predictions in each province, we followed former initiatives (e.g. on influenza[14] and dengue[16]) and partitioned the whole clinical cases datasets into two parts. First part was used for calibrating the models and the remaining part (52 weeks) were left aside for out-of-fit prediction. For Riyadh, Macca and Madina prediction periods were, respectively, from weeks 105–156 (7^th^ June, 2015 - 11^th^ June, 2016), weeks 65–116 (11^th^ July, 2015 - 2^nd^ July, 2016), and weeks 63–114 (11^th^ July, 2015 - 2^nd^ July, 2016)(Fig 3). We generated predictions for three major characteristics of the epidemiological cycle similar to previous attempts made for cholera, dengue and influenza[15,41–43] namely: (a) peak week: the week during which the maximum number of clinical cases occurred in a season (comprising 52 weeks); (b) peak maximum: the number of cases occurring at the peak week, and (c) season totals: the total number of cases in the entire season. Prediction for each target variable was made every 4 weeks (i.e. week 0, 4, 8, …, 48), with week 0 corresponding to the first week of the prediction interval. In the case of predictions for week *N*, data up to week *N* was used to fit the model and the trajectory predicted for out-of-fit future weeks *N+1* through week 52 (Fig 3). Indeed, the best models at predicting MERS-CoV incidence differed among regions (Fig 4), highlighting the complexity of the epidemiological situation, particularly in Riyadh. There, where at least there were two strains co-circulating, M2 stands up clearly as the best model function (see bottom panel in Fig 4), in comparison with Macca and Madina, with only one strain (Fig 4 top panels).

### c) Estimation of R_0_, R_C_, and R_H_ and prediction framework

We estimated the basic reproduction number (*R*_*0*_), the community reproduction number (*R*_*C*_), and the hospital reproduction number (*R*_*H*_) for the whole period of data for Riyadh, Macca and Madina (S1–S18 Tables; 44–45). We also estimated weekly values of *R*_*0*_, *R*_*C*_, and *R*_*H*_ for each prediction interval in the three provinces (i.e. weeks 105–156 in Riyadh, weeks 65–116 in Macca and weeks 63–114 in Madina, respectively). The predictions of *R*_*0*_, *R*_*C*_, and *R*_*H*_ are made for each 4-week time interval in the prediction period. The estimate of *R*_*0*_, *R*_*C*_, and *R*_*H*_ during 0^th^ week is obtained using the estimated parameter values for the training period of MERS-CoV data (i.e., in Riyadh week 1–104, in Macca week 1–64 and in Madina week 1–62). Afterwards, we keep on adding 4 data points to the previous data and re-estimate the parameters. These estimated parameters were then plugged into obtain values of *R*_*0*_, *R*_*C*_, and *R*_*H*_ in 4^th^ week to 48^th^ week of the prediction period. Thus in intervals of 4 weeks, we obtain an estimate of *R*_*0*_, *R*_*C*_, and *R*_*H*_. Temporal evolution of *R*_*0*_, *R*_*C*_, and *R*_*H*_ for the three provinces is depicted in S1–S4 Figs, respectively.

**Fig 5:**
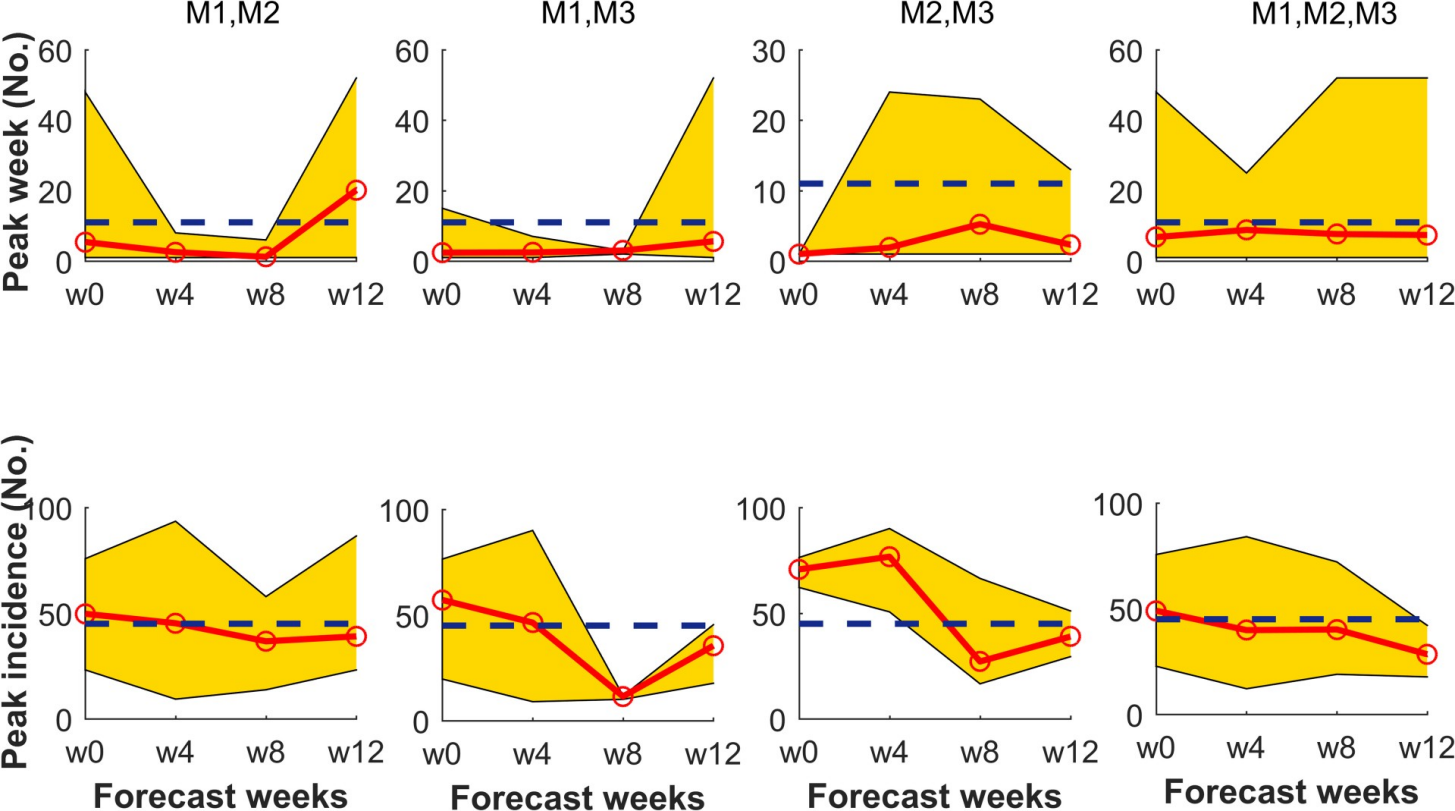
Best fit obtained from a combination of 2-strain models. Each model was aimed at predicting MERS-CoV epidemiological targets (top-peak week (No.), bottom-peak incidence (No.)) in Riyadh. Panels showing peak week and peak incidences are displayed up to the time of occurrence (11th week). Representations of the 2-strain models M1, M2, and M3 are the same as in Fig 2A. Dashed lines are observed values.

Using the model that provides the best prediction for the season total cases in the three provinces (i.e., a 2-strain model with BL incidence for Macca and the 2-strain model with SAT incidence for both Madina and Riyadh), we predicted the total number of cases in the following year. Using the parameter estimates in the last prediction point (i.e. 48^th^ week) of the current season, we draw 1000 samples. Based on these parameter samples, we determined 1000 estimates of the season total cases in the next year[43]. We then defined a large outbreak in the forthcoming season in a particular region, as those events when cases exceeded the total number of cases in the previous season by more than one standard deviation (see Fig 5). Thus, the probability of a large outbreak (P_l_) in those provinces isdetermined as the ratio of samples that exceed the total number of cases in a season divided by the total number of samples. We also considered the case when two strains of MERS-CoV may be co-circulating in the human population in Saudi Arabia. We also simulated the situation when one strain is assumed to be more active with a higher transmission rate, whilst the other is much less transmissible among individuals in the different provinces of Saudi-Arabia[37].Although we have considered two strains circulating in the community setting, we do not distinguish among strains in hospital premises. This is due to the lack of strain specific data in hospital settings. However, we are able to distinguish the contribution of infection from Community and Hospital setting by estimating the Community reproduction number (R_C_) and the Hospital reproduction number (R_H_). However, to account for this effect, we propose three different forces of infection (BL, NM and SAT) to model the MERS transmission in the three provinces. Saturated (SAT) functions describe “Crowding effects” in disease transmission, whereas Non-monotone (NM) functions are used to incorporate”psychological effects”. As the CFR in MERS-CoV is about 40%, it is easily expected that awareness in front of a MERS-CoV situation may play an important role during an epidemic[44–46]. Thus a model with such a crowding effect might seem a priori more realistic in comparison to the bilinear (BL) transmission (see also Fig 2). The crowding effect in disease transmission[38,39] can also be interpreted as the behavioral change of susceptible individuals when the number of infected individuals increases (Fig 2). This fact causes a lower number of new infections when a large number of infected individuals are already present in the community. This behavioral change in the susceptible population may occur due to, for instance, public health awareness campaigns and alerts raised by local or international authorities. The psychological effect is somewhat similar to the crowding effect, but with the effect of fear being added to the awareness. The psychological effect is stronger as the transmission rate decreases more rapidly with an increasing number of infected individuals (Fig 2) [40–42]. Recently, Kucharski et al.[35] suggested that MERS-CoV transmission is over dispersed and hence outbreaks can include super-spreading events. In a similar study, Nishiura et al.[33] concluded that super-spreaders who visited multiple healthcare facilities drove up the epidemic by generating larger number of secondary cases. Therefore, we also consider the role of potential super-spreaders in the context of a 2-strain model with BL incidence function.

## Results and discussion

Estimates of the transmission rates for the 2-strain model with BL incidence suggest that MERS-CoV transmission in the three locations is dominated by community and hospital transmission (S1 Table to S9 Table). The former statement is in good agreement with a previous study that suggested that MERS-CoV infections are essentially produced through both hospital and community based human-human transmission[2]. This fact is well reflected in the estimates of transmission rates obtained from the other two 2-strain models (e.g. NM and SAT incidence functions) for the three locations (S1–S9 Tables). Estimates of the parameter *θ* (a measure of variability within the two strains) in Riyadh and Macca (see S1–S3 Tables) suggest that both strains are currently active in these provinces (albeit strain 2 contributes with only 3% - 50% to community human-human transmission). In Madina instead, a majority of the models (BL and NM) point to only one active strain,with strain 2 contributing less than 1% to community human-human transmission, see S1–S9 Tables.

For each particular region in this study, two strain models are compared among themselves and also with regard to their single-strain counterparts in order to determine the best model (i.e. single, two-strain or a combination of other two-strain models). Results show that in the three locations, 2-strain models provide a better fitting to cumulative cases in comparison to their single strain counterparts (Fig 2). AIC and BIC values for the 2-strain models suggest that the best model is region specific (best model for Riyadh is a 2-strain model with BL incidence, whereas in Macca and Madina the best models are both the model with a NM incidence function and the one incorporating super-spreaders, respectively; see S19 Table). We also compare our 2-strain models with a 2-strain model with variable Zoonotic transmission (SI Eq. A2). Comparing AIC and BIC values, we found that the models with variable Zoonotic transmission (SI Eq. A2) did not improve all the previous results (see S19 Table). Therefore, the best model among all 2-strain models cannot be determined solely from their goodness-of-fit comparison. For this reason, we additionally compared all the two-strain models on the basis of their respective prediction skills. Forecasts for the three models in Riyadh suggest that, a majority of the times, the 2-strain model with SAT incidence can better predict the three targets in comparison to the other two competing 2-strain models (e.g. note that for SAT the average of the mean absolute error, MAE, for peak week is 11.6, whereas for peak maximum is 24.83 and for season totals 55.62) (Table 2A). For comparison, we also provide predictions in the province of Riyadh of the three targets using the model with super-spreaders. However, prediction with super-spreaders did not improve at all the former results obtained for Riyadh (see Table 2A). For Madina, both the peak week and season totals are better predicted by the same SAT 2-strain model (i.e. MAE average for peak week being 14.5 and 9.33 for season totals; see also S22 Table). However, when predicting the peak maximum in Madina, clearly the best model is the 2-strain model with the NM incidence function (i.e. MAE average for peak maximum being 2.62; see also S22 Table). For Macca, except for the peak week, the other two targets, namely peak maximum and season totals are better predicted with the 2-strain model with BL incidence (i.e. MAE average of 7.08 for peak maximum and of 63.42 for season totals, respectively; see S22 Table). For peak week in Macca, again a better prediction is achieved by the 2-strain model with SAT incidence (i.e. MAE average for peak week being 23.9, see S22 Table).

**Table 2:** (A)Average predictions [Simple average of Mean Absolute Errors (MAE)] over all forecasts of the three 2-strain models and their different combinations for the Riyadh province. (B)Estimated values of the Basic reproduction number (R_0_), the Hospital reproduction number (R_H_), and the Community reproduction number (R_C_)for the three provinces of Saudi Arabia using best model (two strain). The best two strain MERS model is with saturated incidence. The data are given as Mean (95% CI). *The MAE for the three models and their combinations during point prediction of peak week and peak incidence were calculated up-to the peak of the prediction season (11th Week) of the Riyadh province.

A Observed values (Data)	Peak week* (weeks)	Peak maximum* (cases)	Season totals (cases)
	11	45	230
Individual model forecasts			
M1	22.8 [19.5]	18.4 [32.94]	478.80 [69.33]
M2	28.7 [19.7]	16.5 [26.25]	216.60 [75.87]
M3	22.1 [11.6]	19.2 [24.83]	222.12 [55.62]
M1 with Superspreaders	44.1 [34.7]	68.9 [32.4]	533.71 [194.5]
Average model forecasts			
M1-M2	7.3 [12.1]	42.67 [18.06]	299.73 [69.92]
M1-M3	3.4 [9.5]	37.63 [22.19]	296.18 [68.31]
M2-M3	2.6 [9.4]	53.37 [21.74]	270.47 [42.79]
M1-M2-M3	7.7 [9.6]	39.37 [17.21]	306.90 [77.41]
B Provinces	R0 [Mean (95% CI)]	RH [Mean (95% CI)]	RC [Mean (95% CI)]
Riyadh	2.0706 [2.0629–2.0763]	2.0508 [2.0442–2.0543]	0.5657 [0.5587–0.5709]
Macca	4.5716 [2.8781–6.5899]	2.7239 [0.4281–4.9401]	2.0977 [0.1256–5.7307]
Madina	5.0661 [2.5264–9.3814]	4.7808 [0.7408–9.3132]	1.6506 [0.2116–2.9661]

When comparing the three two strain models to their single-strain counterparts, composites of the mean absolute error (MAE) in Riyadh (Table 2A, and S23 Table) suggest that 2-strain models always outperform the single strain versions in predicting the three targets *(*i.e. peak week, peak maximum, and season totals). Averages of MAE for peak week, peak maximum and for season totals are 11.6, 24.83 and 55.62, respectively. This fact reinforces our earlier inference, confirming that there is more than one strain currently active in Riyadh. Among the single-strain models in Riyadh, the model with SAT incidence is found to have a better predictive capacity for the three targets in comparison to the other two single-strain models (S23 Table). In Macca instead, the single-strain model with SAT incidence provides better predictions of the peak week, the peak maximum and seasontotals in comparison to all the other models (see for instance MAE values in S22 Table; and values obtained for 1-strain and 2-strain models in S22 Table). Conversely, in Madina, both 1-strain and 2-strain models with SAT incidence provide a similar performance in predicting the three targets (e.g. MAE for peak week 16.7 in comparison to 14.5, for peak maximum of 3.33 compared to 3.6 and for season totals of 5.87 against 9.33; see S22 and S23 Tables). The latter may likely represent the best choice of models to form the basis of a future early-warning system for MERS-CoV prediction in the region.

The best model, selected on the basis of its capacity to derive skillful predictions, is the model with the SAT incidence function. We used this model to estimate the basic reproduction number, R_0_, the community reproduction number, R_C_, and the hospital reproduction number, R_H_, for the whole period of data available in those three provinces (Table 2B and S20 Table). In all three provinces, R_0_ is estimated to be always greater than unity. In all three provinces, R_H_ is found out to be larger than R_C_. This implies, MERS-CoV transmission triggered from the hospital setting (see Table 2B). These estimates agree with some previous values reported in the literature[4,17–18], while being a result well supported by other R_0_ estimates[29–31, 33]. However, previous modeling attempts to model Saudi Arabia MERS-CoV clinical incidence used only a 1-strain model with BL transmission[4]. In our case, versions of R_0_ were also estimated from our BL 1-strain model in those same three provinces (see S21 Table) and they are in good agreement with the previous values provided by Chowell et al.[4]. At the same time, though, 1-strain model show less predictive capacity than their similar two-strain counterparts. To further verify the robustness of these estimates, the temporal evolution of R_0_, R_C_, and R_H_ are displayed for different time intervals in the three provinces (S1–S4 Figs). Considering the best model configurations, we additionally computed the temporal evolution of R_0_, R_C_, and R_H_ in the three provinces. Temporal changes in R_0_ (S1 Fig) indicate that in most of the predicted weeks, R_0_ stays well above the epidemic threshold (R_0_ = 1). This fact is well established from the temporal evolution of R_C_, and R_H_ in those three provinces (S1 and S2 Figs).

**Fig 6:**
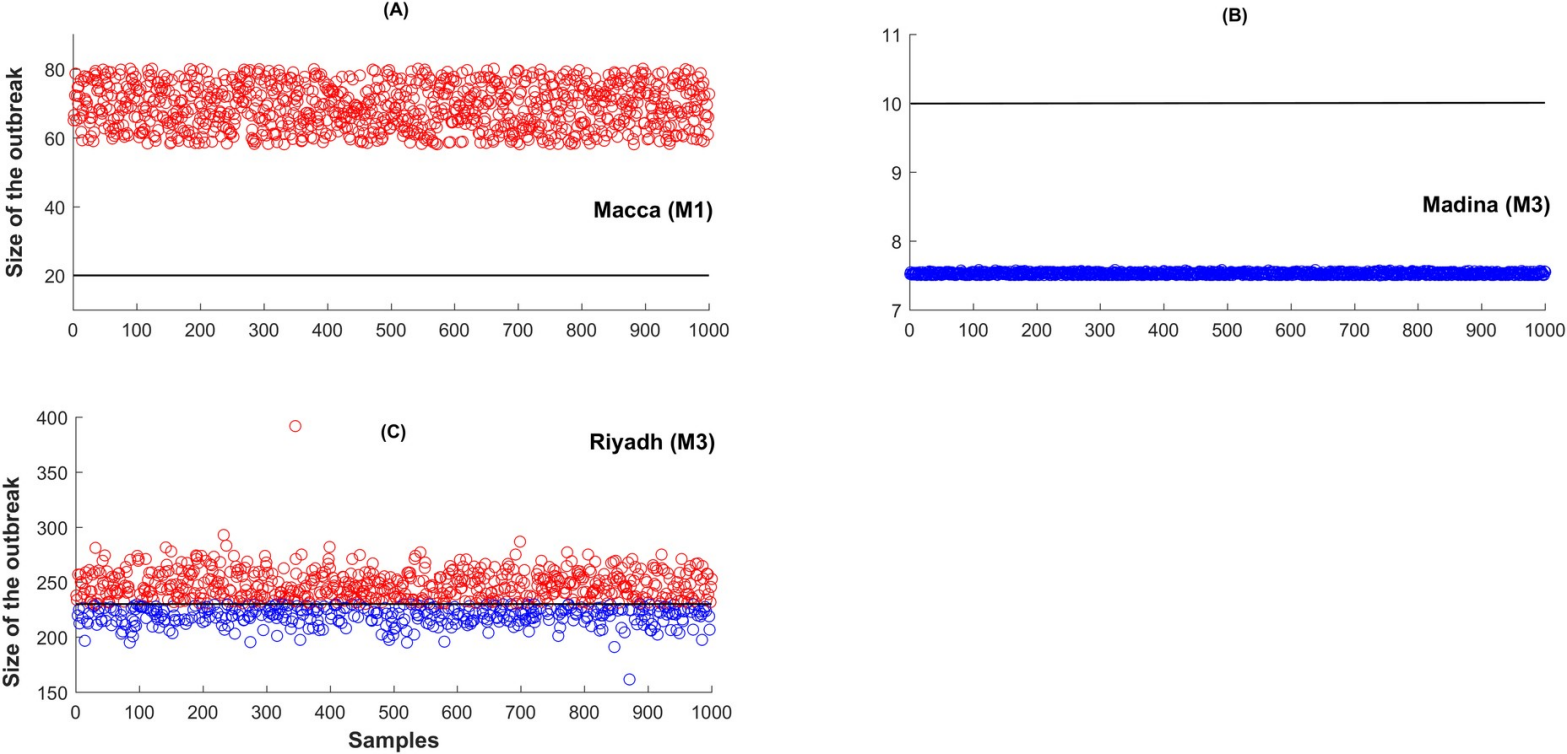
Out-of-fit prediction of large outbreaks in Macca, Madina and Riyadh in July 2016 to July 2017. Large outbreak size (red circles) are defined as those samples which exceeds previous year (July 2015 to July 2016) total season cases. Blue circles denote those samples that fall below previous year total cases. The black line denotes total cases during July 2015 to July 2016 of the data. M1: two strain model with bilinear incidence, M3: two strain model with saturated incidence.

**Fig 7:**
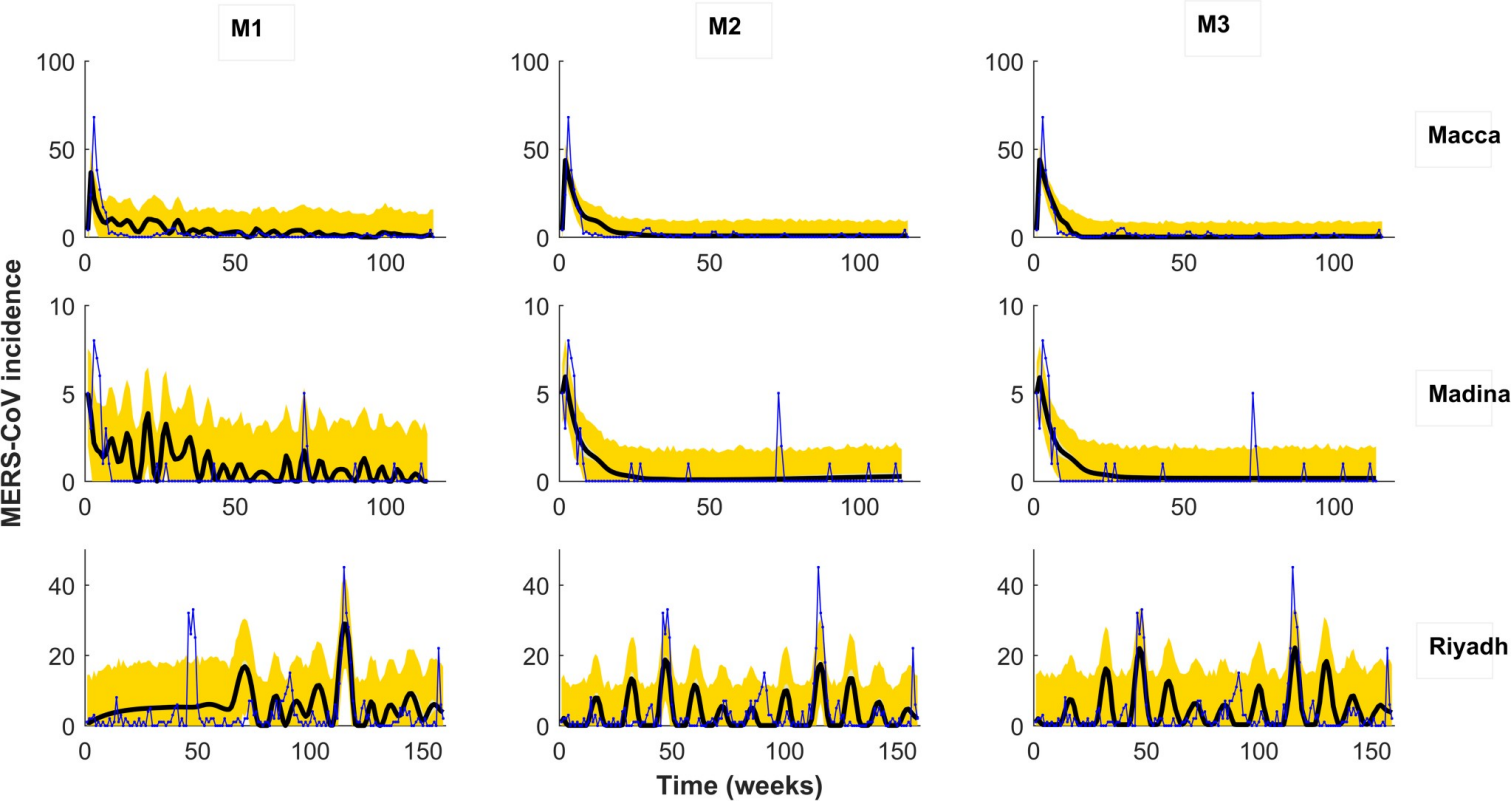
Single-strain model fitting to new MERS-CoV cases. Here M1: single strain model with BL force of infection, M2: single strain model with non-monotone incidence, and M3: single strain model with saturated incidence. Solid black curve represents model solution and yellow region denotes 95% confidence interval for predictions.

**Fig 8:**
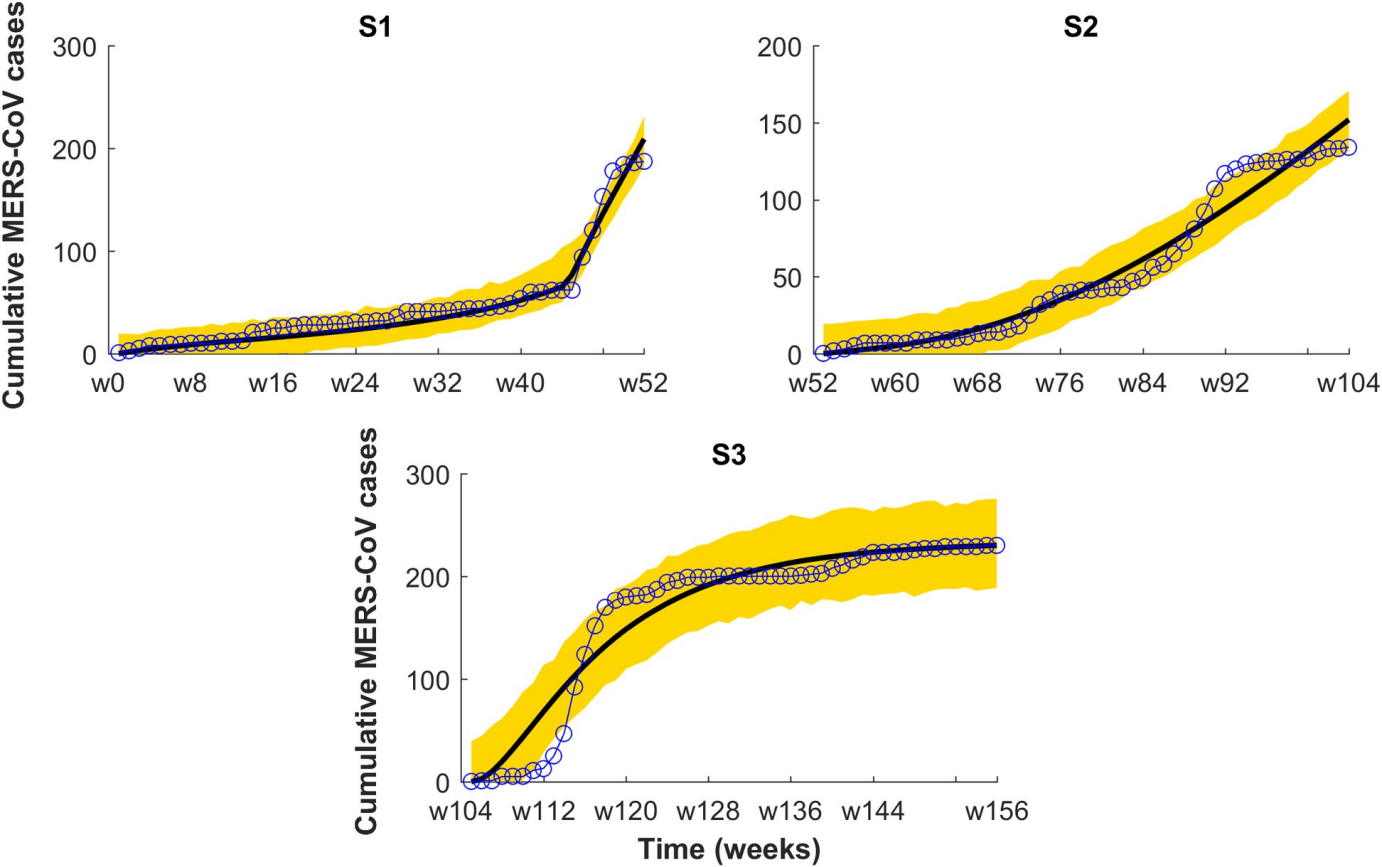
Season-wise model fitting to cumulative cases of Riyadh from July 6, 2013 to June 28, 2016. (Model fitted is a 2-strain model with saturated incidence. One year appears in each panel S1 to S3(S1 = S2 = S3 = 52 weeks). Line and circle line refers to observations and 95%confidence interval for cumulative predictions denoted by the yellow region.

As the basis for an operational EWS for the region, we predicted the total number of cases in the out-of-fit interval covering July 2016 to July 2017. We used the best individual model for the three provinces among all 2-strain and single strain models developed. Figs 6 and 7 and Table 3 clearly indicate the 2016–2017 season might be ripe for a larger outbreak in Macca and Riyadh. Instead, in Madina the likelihood of suffering a larger outbreak is very low (Fig 6 and Table 3). Fig 7 displays the single-strain model fitting to new MERS-CoV cases. Here M1 refers to the single strain model with BL force of infection, M2 to the single strain model with non-monotone incidence, and M3 to the single strain model with saturated incidence. Solid black curve represents model solution and yellow region denotes 95% confidence interval for predictions. Simulations for both Macca and Riyadh reflect quite appropriately the dynamics displayed in observations (Fig 7). Fig 8 shows All-season’s predictions for the three consecutive years from July 6, 2013 to June 28, 2016, with each interval of 52 weeks fitted shown in panels S1-S3.

**Table 3:** Hindcast prediction of season total MERS-CoV cases for July 2016 to July 2017 and probability of large outbreak. Prediction based upon the best two-strain model (in terms of season totals forecast) for the three provinces of the Saudi-Arabia.

Two strain models	MERS-CoV cases for July 2015 –July 2016	Predicted cases of MERS-CoV for the period July 2016 –July 2017 (Mean (95% CI))	Probability of larger outbreak ((No. of samples above 2015/2016 cases)/(total samples))
Riyadh (M3)	230	248.1724 (213.7383–268.26)	0.7150
Macca (M1)	20	69.0813(59.7604–78.9765)	1
Madina (M3)	10	7.5268 (7.5087–7.5598)	0

According to WHO reports, 249 MERS-CoV cases including 75 deaths (CFR 30%) were reported from Saudi Arabia between July 2016 and July 2017[44]. In the aforementioned period, at least 108 new cases[44] were reported from Riyadh province and at least 5 cases were reported from Madina. These values indicate that a larger epidemic did not occur in the last season (2016–2017), the risk for a larger one in the coming seasons still remains high.

### Conclusions

Up to July 2018, 2237 new cases were reported, with 1861 only in KSA and 793 deaths[45](CFR 35.5%). This is an alarming situation as previous predictions on MERS-CoV had instead suggested that MERS-CoV might not sustain as an epidemic in the Arabian Peninsula. The WHO report[44] suggests that MERS-CoV is still a relatively rare disease about which the medical personnel in health-care facilities have low awareness. Globally, MERS-CoV awareness is limited and because symptoms of MERS-CoV infection are non-specific, initial cases can be sometimes easily missed. With improved compliance in infection prevention and control, namely by stricter adherence to the standard precautions at all times, human-to-human transmission in health-care facilities can be reduced and even possibly eliminated with additional use of transmission-based precautions. In that regard, predictive mathematical models can help strengthen our understanding of both MERS-CoV transmission and control.

In this study, we addressed the capacity of predictive mathematical models based on two-strain MERS-CoV configurations having different transmission functions. The models differed from each other in their force of infection and in how they cope with heterogeneity in transmission. Estimates of transmission rates suggest that community and hospital transmission are dominant in the case of 2-strain models in Riyadh, Macca and Madina. The majority of the 2-strain models suggest that MERS-CoV transmission is dominated by community and hospital human-human transmissions, a fact that reflects the actual transmission scenario in Saudi Arabia [2–4]. Estimates of the parameter that measures transmission diversity between the two strains in the three provinces suggest that two strains are only active in Riyadh. This opposite trend in Riyadh in comparison to the other two provinces may be due to the fact that Riyadh is the capital city with good large health care facilities and a majority of the MERS-CoV patients in Saudi Arabia come to Riyadh for treatment[2]. These patients may therefore carry different MERS strains, ultimately leading to multiple strains being presently co-circulating in Riyadh. Similarly, Cotten et al.[37] found that ancestors of most of the viral clades originated from Riyadh.

We compared among the 2-strain models according to their predictive performance with regard to three targets (i.e. peak week, peak maximum and season totals). Our results suggest that among the three 2-strain models, the model with SAT incidence provides consistently skillful predictions and may be used to date as the best predictive model for MERS-CoV in Riyadh. Riyadh iswhere most of the MERS-CoV cases occur, while for the provinces of Macca and Madina, with lower reported MERS cases, it is difficult to determine the best model among the three 2-strain models. This fact justifies our earlier finding that in Riyadh two different strains are currently active and therefore the performance of the 2-strain models is better there. As per our results for Macca and Madina, only one dominant strain is active in those provinces. Therefore, predictions based on single strain models are there more appropriate. Our results also suggest that among the single strain models, those with SAT incidence always accurately predict the three targets for these two provinces. Thus, a dynamical MERS model considering this crowding effect is the most appropriate configuration to cope with the nature of MERS-CoV transmission.

We estimated R_0_ using the best 2-strain model in Riyadh and estimates are in good agreement with the findings of Majumdar et al. [28].The finding that R_0_ is most of the time above 1 (S1 Fig) is well supported by some previous estimates in literature[28–30,32]. Lower contribution of community transmission in R_0_ (See Table 2B) in Riyadh and Macca suggests that MERS-CoV transmission is triggered from hospital settings in those provinces. Most interestingly, in some forecasted periods, R_0_ attains large values, a fact that denotes that a rapid propagation among the susceptible population is indeed possible. More worrisome is the range of values into which R_0_ moves, most of the time above 1 and below 2,5, a fact that makes it a dangerous infection in terms of silent and constant potential population spread. Community reproduction number R_C_ well above unity (see S4 Fig) in most of the predictive weeks indicate that in a near future a large outbreak may be possible in those provinces. Out-of-fit predictions for the next season totals suggest that there is a high possibility of larger outbreaks in Macca and Riyadh. However, our results instead indicate that there is a very low possibility of larger outbreaks in Madina. The fact that this outbreak did not happen in 2017–2018 does not preclude what may occur in the forthcoming seasons. Under such a scenario, authorities and international health agencies should prepare and actively work towards the prompt implementation of cheap albeit efficient computational platforms ready to assist in the simulation of how a potential outbreak might evolve in the region. More so, given the high probability that another large MERS-CoV outbreak occurs in the Arabian Peninsula or nearby countries. Migration may also play a major role for increased transmission in the provinces of Saudi Arabia and this feature should be properly accounted for in future model configurations. In summary, our findings suggest that in a majority of provinces a single MERS-CoV strain is currently active, conversely to the situation in Riyadh. However, in the near future, it is also possible that more general MERS-CoV transmission occurs from multiple strains in other provinces of Saudi Arabia.

## Supporting information

S1 Table Estimated parameters for Model-(A) with bilinear incidence for the Riyadh province. (DOCX)

S2 Table Estimated parameters for Model-(A) with non-monotone incidence for the Riyadh province. (DOCX)

S3 Table Estimated parameters for Model-(A) with saturated incidence for the Riyadh province. (DOCX)

S4 Table Estimated parameters for Model-(A) with bilinear incidence for the Mecca province. (DOCX)

S5 Table Estimated parameters for Model-(A) with non-monotone incidence for the Mecca province. (DOCX)

S6 Table Estimated parameters for Model-(A) with Saturated incidence for the Mecca province. (DOCX)

S7 Table Estimated parameters for Model-(A) with bilinear incidence for the Madina province. (DOCX)

S8 Table Estimated parameters for Model-(A) with non-monotone incidence for the Madina province. (DOCX)

S9 Table Estimated parameters for Model-(A) with saturated incidence for the Madina province. (DOCX)

S10 Table Estimated parameters for the Model (B) with bilinear incidence for the Riyadh province. (DOCX)

S11 Table Estimated parameters for the Model (B) with non-monotone incidence for the Riyadh province. (DOCX)

S12 Table Estimated parameters for the Model (B) with saturated incidence for the Riyadh province. (DOCX)

S13 Table Estimated parameters for the Model (B) with bilinear incidence for the Macca province. (DOCX)

S14 Table Estimated parameters for the Model (B) with non-monotone incidence for the Macca province. (DOCX)

S15 Table Estimated parameters for the Model (B) with saturated incidence for the Macca province. (DOCX)

S16 Table Estimated parameters for the Model (B) with bilinear incidence for the Madina province. (DOCX)

S17 Table Estimated parameters for the Model (B) with non-monotone incidence for the Madina province. (DOCX)

S18 Table Estimated parameters for the Model (B) with saturated incidence for the Madina province. (DOCX)

S19 Table Multi-model inference quantities (AIC and BIC) for three two-strain and single-strain models. Model with smallest AIC and BIC value are given in bold.
(DOCX)

S20 Table Estimated values of the Basic reproduction number (R0), the Hospital reproduction number (RH), and the Community reproduction number (RC), for the three provinces of Saudi Arabia for the two strain Model-1 and Model-2 (Equation (A) with bilinear incidence and non-monotone incidence). The data is given in the format (Mean [95%CI]).
(DOCX)

S21 Table Comparison of estimated values of the Basic reproduction number (R0), the Hospital reproduction number (RH), and the Community reproduction number (RC), for the three provinces of Saudi Arabia for the single strain Model-1, Model-2 and Model-3 (Equation (B) with bilinear, non-monotone and saturated incidence). The data is given in the format (Mean [95%CI]).
(DOCX)

S22 Table Average predictions [Simple average of Mean Absolute Errors (MAE)] obtained over all the prediction weeks using Model -(A) with different incidence functions. Model -1 represents Model-(A) with bilinear incidence function. Model -2 represents Model-(A) with non-monotone incidence and Model -3 represents Model -(A) with saturated incidence.
(DOCX)

S23 Table Aver.age predictions [Simple average of Mean Absolute Errors (MAE)] obtained over all the prediction weeks using a single-strain Model—(B) with different incidence functions. Model -1 represents Model-(B) with bilinear incidence function. Model -2 represents Model-(B) with non-monotone incidence and Model-3 represents Model-(B) with saturated incidence.
(DOCX)

S24 Table Estimated parameters for Model-(A1) for Riyadh. (DOCX)

S25 Table Estimated parameters for Model-(A1) for Macca. (DOCX)

S26 Table Estimated parameters for Model-(A1) for Madina. (DOCX)

S27 Table Estimated parameters for the Model (B1) for Riyadh. (DOCX)

S28 Table Estimated parameters for the Model (B1) for Macca. (DOCX)

S29 Table Estimated parameters for the Model (B1) for Madina. (DOCX)

S1 Fig Temporal Evolution of R0 using the best predicted 2-strain model (Saturated incidence) in three provinces; Riyadh, Macca and Madina. R0 is estimated over different forecast weeks (0, 4, 8, …, 48).
(DOCX)

S2 Fig Temporal Evolution of Hospital reproduction number (RH) using the best predicted 2-strain model (Saturated incidence) in three provinces; Riyadh, Macca and Madina. RH is estimated over different forecast weeks (0, 4, 8, …, 48). Dotted line represents the threshold of the epidemic potential (RH = 1).
(DOCX)

S3 Fig Temporal Evolution of Community reproduction number (RC) using the best predicted 2-strain model (Saturated incidence) in three provinces; Riyadh, Macca and Madina. RC is estimated over different forecast weeks (0, 4, 8, …, 48). (DOCX)

S4 Fig Model simulations fitted to accumulated MERS-CoV clinical cases in Macca, Madina and Riyadh. (DOCX)
