# Supplementary material for: A realistic two-strain model for MERS-CoV infection uncovers the high risk for epidemic propagation
Source: PLoS Negl Trop Dis. 2020 Feb 14;14(2):e0008065. doi: 10.1371/journal.pntd.0008065 (PMC7046297; doi:10.1371/journal.pntd.0008065)
Supplement: S1 Table — (DOCX) [file pntd.0008065.s001.docx]

| Parameters | Mean | 95% CI |
| --- | --- | --- |
| β_1_ | 73.7757 | 37.8825 - 99.1158 |
| $\theta$ | 0.1831 | 0.0132 – 0.4657 |
| $\rho$ | 0.6167 | 0.1661 - 0.9847 |
| β_2_ | 2.3006 | 0.1615-7.0773 |
| β_3_ | 2.9602 | 0.1087 - 8.4076 |
| $p_{1}$ | 0.4301 | 0.0359 - 0.9348 |
| $p_{2}$ | 0.3632 | 0.0149 - 0.9647 |
| $c_{1}$ | 1.3593 | 0.5513 - 2.2656 |
| $c_{2}$ | 0.7573 | 0.0447 - 1.7394 |
| E_1_(0) | 5.2040e-4 | 4.065e-5 - 0.0014 |
| E_2_(0) | 0.2470 | 0.0207 - 0.5663 |
| A_1_(0) | 0.0823 | 0.0027 - 0.2259 |
| A_2_(0) | 0.1304 | 0.0057 - 0.3354 |
| I_1_(0) | 0.0028 | 1.8366e-4 - 0.0087 |
| I_2_(0) | 0.0642 | 0.0035 - 0.2144 |
|  |  |  |

S1 Table: Estimated parameters for Model-(A) with bilinear incidence for the Riyadh province
