# Supplementary material for: A realistic two-strain model for MERS-CoV infection uncovers the high risk for epidemic propagation
Source: PLoS Negl Trop Dis. 2020 Feb 14;14(2):e0008065. doi: 10.1371/journal.pntd.0008065 (PMC7046297; doi:10.1371/journal.pntd.0008065)
Supplement: S2 Table — (DOCX) [file pntd.0008065.s002.docx]

| Parameters | Mean | 95% CI |
| --- | --- | --- |
| β_1_ | 4.3534 | 0.1010 - 13.0442 |
| $\theta$ | 0.0311 | 0.0072 - 0.0615 |
| $\rho$ | 0.0096 | 5.7867e-4 - 0.0214 |
| β_2_ | 9.4884 | 0.9454 - 13.7752 |
| β_3_ | 0.3532 | 0.0109 - 1.5433 |
| $p_{1}$ | 0.4686 | 0.0125 - 0.9695 |
| $p_{2}$ | 0.6889 | 0.1675 - 0.9939 |
| $c_{1}$ | 0.0271 | 0.0020 - 0.0659 |
| $c_{2}$ | 0.1640 | 0.0174 - 0.4487 |
| E_1_(0) | 1.7872 | 0.2054 - 4.0584 |
| E_2_(0) | 0.7186 | 0.0279 - 2.2567 |
| A_1_(0) | 16.3460 | 1.3200 - 29.4081 |
| A_2_(0) | 10.2821 | 0.4217 - 27.6685 |
| I_1_(0) | 0.4284 | 0.0392 - 1.0193 |
| I_2_(0) | 0.7326 | 0.4807 - 1.0240 |
| α_1_ | 285.6211 | 26.0462 – 492.7555 |
| α_2_ | 256.7281 | 29.3088 - 476.5971 |
|  |  |  |

S2 Table: Estimated parameters for Model-(A) with non-monotone incidence for the Riyadh province
