# Supplementary material for: A realistic two-strain model for MERS-CoV infection uncovers the high risk for epidemic propagation
Source: PLoS Negl Trop Dis. 2020 Feb 14;14(2):e0008065. doi: 10.1371/journal.pntd.0008065 (PMC7046297; doi:10.1371/journal.pntd.0008065)
Supplement: S3 Table — (DOCX) [file pntd.0008065.s003.docx]

| Parameters | Mean | 95% CI |
| --- | --- | --- |
| β_1_ | 3.3927 | 3.3881 - 3.3960 |
| $\theta$ | 0.0350 | 0.0311 - 0.417 |
| $\rho$ | 0.1360 | 0.1301 - 0.1406 |
| β_2_ | 8.2433 | 8.2388 - 8.2455 |
| β_3_ | 6.1331 | 6.1309 - 6.1362 |
| $p_{1}$ | 0.0017 | 1.5775e-5 - 0.0049 |
| $p_{2}$ | 0.0898 | 0.0891 - 0.0909 |
| $c_{1}$ | 0.4447 | 0.4440 - 0.4458 |
| $c_{2}$ | 0.2600 | 0.2553 - 0.2647 |
| E_1_(0) | 1.9897 | 1.9846 - 1.9925 |
| E_2_(0) | 0.6496 | 0.6479 - 0.6525 |
| A_1_(0) | 8.9593 | 8.9522 - 8.9634 |
| A_2_(0) | 5.5422 | 5.5393 - 5.5453 |
| I_1_(0) | 0.0895 | 0.0870 - 0.0911 |
| I_2_(0) | 0.7578 | 0.7550 - 0.7620 |
| α_1_ | 463.7338 | 463.7291 - 463.7383 |
| α_2_ | 445.5564 | 445.5518 - 445.5637 |
|  |  |  |

S3 Table: Estimated parameters for Model-(A) with saturated incidence for the Riyadh province
