# Supplementary material for: A realistic two-strain model for MERS-CoV infection uncovers the high risk for epidemic propagation
Source: PLoS Negl Trop Dis. 2020 Feb 14;14(2):e0008065. doi: 10.1371/journal.pntd.0008065 (PMC7046297; doi:10.1371/journal.pntd.0008065)
Supplement: S4 Table — (DOCX) [file pntd.0008065.s004.docx]

| Parameters | Mean | 95% CI |
| --- | --- | --- |
| β_1_ | 16.0023 | 14.1405 - 17.5228 |
| $\theta$ | 4.5550e-6 | 1.7825e-7 - 1.7411e-5 |
| $\rho$ | 0.0029 | 7.4133e-5 - 0.0126 |
| β_2_ | 1.2752e-4 | 6.5911e-6 - 4.2872e-4 |
| β_3_ | 0.3587 | 0.0057 - 1.3211 |
| $p_{1}$ | 0.5402 | 0.0274 - 0.9760 |
| $p_{2}$ | 0.0654 | 9.5447e-4 - 0.2173 |
| $c_{1}$ | 5.6784e-6 | 5.7347e-7 - 2.337e-5 |
| $c_{2}$ | 7.5697e-6 | 3.0727e-7 - 2.6699e-5 |
| E_1_(0) | 0.0099 | 2.9452e-4 - 0.0262 |
| E_2_(0) | 9.1623e-5 | 1.1632e-5 - 3.0541e-4 |
| A_1_(0) | 0.8103 | 0.1169 -2.7490 |
| A_2_(0) | 17.1146 | 1.7086 - 29.2931 |
| I_1_(0) | 8.2180e-5 | 2.4213e-6 - 2.8926e-4 |
| I_2_(0) | 11.7745 | 10.5761 - 12.9099 |

S4 Table: Estimated parameters for Model-(A) with bilinear incidence for the Mecca province
