# Supplementary material for: A realistic two-strain model for MERS-CoV infection uncovers the high risk for epidemic propagation
Source: PLoS Negl Trop Dis. 2020 Feb 14;14(2):e0008065. doi: 10.1371/journal.pntd.0008065 (PMC7046297; doi:10.1371/journal.pntd.0008065)
Supplement: S5 Table — (DOCX) [file pntd.0008065.s005.docx]

| Parameters | Mean | 95% CI |
| --- | --- | --- |
| β_1_ | 19.7848 | 17.0434 - 23.4033 |
| $\theta$ | 0.5008 | 0.0211 - 0.9812 |
| $\rho$ | 0.4790 | 0.0205 - 0.9740 |
| β_2_ | 0.0097 | 2.4e-4 - 0.0409 |
| β_3_ | 0.8710 | 0.0425 -2.5183 |
| $p_{1}$ | 0.5322 | 0.0278 - 0.9764 |
| $p_{2}$ | 0.5138 | 0.0255 - 0.9834 |
| $c_{1}$ | 4.3567e-5 | 2.2444e-7 - 3.2613e-4 |
| $c_{2}$ | 2.8197e-5 | 1.0132e-6 - 1.7167e-4 |
| E_1_(0) | 2.7349e-4 | 6.3948e-6 - 0.0019 |
| E_2_(0) | 0.0033 | 9.7151e-5 - 0.023 |
| A_1_(0) | 1.2021e-4 | 4.5756e-6 - 8.0642e-4 |
| A_2_(0) | 16.4546 | 1.2525 - 29.4546 |
| I_1_(0) | 5.1118 | 0.1938 - 10.6712 |
| I_2_(0) | 5.6312 | 0.3961 -10.7609 |
| α_1_ | 294.0261 | 59.0587 - 488.9904 |
| α_2_ | 234.5921 | 10.9866 - 488.9648 |

S5 Table: Estimated parameters for Model-(A) with non-monotone incidence for the Mecca province
