# Supplementary material for: A realistic two-strain model for MERS-CoV infection uncovers the high risk for epidemic propagation
Source: PLoS Negl Trop Dis. 2020 Feb 14;14(2):e0008065. doi: 10.1371/journal.pntd.0008065 (PMC7046297; doi:10.1371/journal.pntd.0008065)
Supplement: S6 Table — (DOCX) [file pntd.0008065.s006.docx]

| Parameters | Mean | 95% CI |
| --- | --- | --- |
| β_1_ | 7.6085 | 0.5027 - 17.6490 |
| $\theta$ | 0.4966 | 0.0187 - 0.9531 |
| $\rho$ | 0.4941 | 0.0263 -0.9717 |
| β_2_ | 12.2035 | 1.9075 - 19.9267 |
| β_3_ | 0.7437 | 0.0326 - 1.6771 |
| $p_{1}$ | 0.5512 | 0.0262 -0.9763 |
| $p_{2}$ | 0.5305 | 0.0238 - 0.9676 |
| $c_{1}$ | 1.2628e-4 | 8.2445e-6 - 7.3052e-4 |
| $c_{2}$ | 9.1465e-5 | 5.1582e-6 - 4.8912e-4 |
| E_1_(0) | 0.0085 | 6.6942e-4 - 0.0363 |
| E_2_(0) | 3.9272e-7 | 1.5233e-8 - 1.1920e-6 |
| A_1_(0) | 15.5323 | 0.6190 - 29.0556 |
| A_2_(0) | 14.4214 | 0.5871-28.7631 |
| I_1_(0) | 0.3286 | 0.0155 - 0.8680 |
| I_2_(0) | 10.3942 | 8.9640-11.9713 |
| α_1_ | 248.2602 | 21.2066 - 488.7495 |
| α_2_ | 302.3010 | 27.6595 - 493.4989 |

S6 Table: Estimated parameters for Model-(A) with Saturated incidence for the Mecca province
