# Supplementary material for: A realistic two-strain model for MERS-CoV infection uncovers the high risk for epidemic propagation
Source: PLoS Negl Trop Dis. 2020 Feb 14;14(2):e0008065. doi: 10.1371/journal.pntd.0008065 (PMC7046297; doi:10.1371/journal.pntd.0008065)
Supplement: S7 Table — (DOCX) [file pntd.0008065.s007.docx]

| Parameters | Mean | 95% CI |
| --- | --- | --- |
| β_1_ | 17.0883 | 15.1841 - 19.9219 |
| $\theta$ | 5.8224e-5 | 3.2256e-6 - 2.2207e-4 |
| $\rho$ | 2.7816e-4 | 9.0570e-6 -7.4710e-4 |
| β_2_ | 2.8748e-4 | 1.0269e-5 - 8.4506e-4 |
| β_3_ | 0.0394 | 8.6962e-4 - 0.1470 |
| $p_{1}$ | 0.4768 | 0.0224 - 0.9780 |
| $p_{2}$ | 0.1130 | 0.0021 -0.4630 |
| $c_{1}$ | 4.6671e-4 | 2.0552e-5 - 0.0016 |
| $c_{2}$ | 0.001 | 5.0097e-5 - 0.0033 |
| E_1_(0) | 0.0011 | 4.7403e-5 - 0.0034 |
| E_2_(0) | 1.5194e-4 | 3.4407e-6 - 5.5410e-4 |
| A_1_(0) | 0.4473 | 0.0219 - 1.4959 |
| A_2_(0) | 15.2219 | 1.1184 - 29.0112 |
| I_1_(0) | 6.3614e-4 | 1.7006e-5 - 0.0021 |
| I_2_(0) | 1.6508 | 1.3978 - 1.9254 |
|  |  |  |

S7 Table: Estimated parameters for Model-(A) with bilinear incidence for the Madina province
