# Supplementary material for: A realistic two-strain model for MERS-CoV infection uncovers the high risk for epidemic propagation
Source: PLoS Negl Trop Dis. 2020 Feb 14;14(2):e0008065. doi: 10.1371/journal.pntd.0008065 (PMC7046297; doi:10.1371/journal.pntd.0008065)
Supplement: S8 Table — (DOCX) [file pntd.0008065.s008.docx]

| Parameters | Mean | 95% CI |
| --- | --- | --- |
| β_1_ | 8.9787 | 0.3802 - 20.7593 |
| $\theta$ | 2.4276e-5 | 1.0807e-6 - 9.5557e-5 |
| $\rho$ | 5.6245e-4 | 3.4816e-5 - 0.0030 |
| β_2_ | 13.0046 | 1.1969-23.7762 |
| β_3_ | 0.1326 | 0.0055 - 0.3952 |
| $p_{1}$ | 0.4826 | 0.0247 - 0.9689 |
| $p_{2}$ | 0.4977 | 0.0205 - 0.9718 |
| $c_{1}$ | 3.5839e-5 | 1.0892e-6 - 2.1893e-4 |
| $c_{2}$ | 6.4818e-5 | 4.4059e-6 -3.6096e-4 |
| E_1_(0) | 2.6576e-5 | 1.8215e-7 - 1.5472e-4 |
| E_2_(0) | 0.0016 | 1.4178e-4 - 0.0060 |
| A_1_(0) | 15.1967 | 0.8128 - 29.3568 |
| A_2_(0) | 16.2072 | 0.9445 - 29.3089 |
| I_1_(0) | 5.7378e-4 | 3.1041e-5 - 0.0032 |
| I_2_(0) | 1.4442 | 1.1740 - 1.7146 |
| α_1_ | 267.7104 | 19.0812 - 487.4961 |
| α_2_ | 305.0045 | 74.9034 - 490.3059 |
|  |  |  |

S8 Table: Estimated parameters for Model-(A) with non-monotone incidence for the Madina province
