# Supplementary material for: A realistic two-strain model for MERS-CoV infection uncovers the high risk for epidemic propagation
Source: PLoS Negl Trop Dis. 2020 Feb 14;14(2):e0008065. doi: 10.1371/journal.pntd.0008065 (PMC7046297; doi:10.1371/journal.pntd.0008065)
Supplement: S9 Table — (DOCX) [file pntd.0008065.s009.docx]

| Parameters | Mean | 95% CI |
| --- | --- | --- |
| β_1_ | 12.2178 | 1.5556 - 21.2124 |
| $\theta$ | 0.1178 | 0.0131 - 0.4217 |
| $\rho$ | 0.0359 | 0.0060 - 0.0936 |
| β_2_ | 9.7439 | 1.5114 - 18.9793 |
| β_3_ | 0.0967 | 0.0199 - 0.3601 |
| $p_{1}$ | 0.0033 | 3.6688e-4 - 0.0065 |
| $p_{2}$ | 0.0416 | 0.0066 - 0.1208 |
| $c_{1}$ | 0.0039 | 8.4255e-4 - 0.0093 |
| $c_{2}$ | 0.0025 | 5.9465e-4 - 0.0055 |
| E_1_(0) | 0.0813 | 0.0430 - 0.2174 |
| E_2_(0) | 0.0259 | 0.0065 - 0.0664 |
| A_1_(0) | 0.6852 | 0.1287 - 1.7169 |
| A_2_(0) | 3.0052e-4 | 1.357e-4 - 7.4355e-4 |
| I_1_(0) | 0.9278 | 0.0946 - 1.4148 |
| I_2_(0) | 0.5085 | 0.0663 - 1.3895 |
| α_1_ | 129.9144 | 0.9368 - 474.8810 |
| α_2_ | 378.5509 | 83.2297 - 479.1305 |
|  |  |  |

S9 Table: Estimated parameters for Model-(A) with saturated incidence for the Madina province.
