# Supplementary material for: A realistic two-strain model for MERS-CoV infection uncovers the high risk for epidemic propagation
Source: PLoS Negl Trop Dis. 2020 Feb 14;14(2):e0008065. doi: 10.1371/journal.pntd.0008065 (PMC7046297; doi:10.1371/journal.pntd.0008065)
Supplement: S10 Table — (DOCX) [file pntd.0008065.s010.docx]

| Parameters | Mean | 95% CI |
| --- | --- | --- |
| β_1_ | 0.2496 | 0.1976 – 0.2802 |
| $\rho$ | 0.0060 | 0.0045 – 0.0070 |
| β_2_ | 0.0264 | 0.0168 – 0.0321 |
| β_3_ | 9.3278 | 9.116 – 9.7280 |
| $c_{1}$ | 0.0070 | 0.006 – 0.0085 |
| E(0) | 2.6199 | 2.5616 – 2.653 |
| A(0) | 2.3202 | 1.7678 – 3.2256 |
| I(0) | 0.1295 | 0.1078 – 0.1427 |
|  |  |  |

S10 Table: Estimated parameters for the Model (B) with bilinear incidence for the Riyadh province
