# Supplementary material for: A realistic two-strain model for MERS-CoV infection uncovers the high risk for epidemic propagation
Source: PLoS Negl Trop Dis. 2020 Feb 14;14(2):e0008065. doi: 10.1371/journal.pntd.0008065 (PMC7046297; doi:10.1371/journal.pntd.0008065)
Supplement: S11 Table — (DOCX) [file pntd.0008065.s011.docx]

| Parameters | Mean | 95% CI |
| --- | --- | --- |
| β_1_ | 0.0088 | 0.0006 – 0.0193 |
| $\rho$ | 0.6720 | 0.2098 – 0.9875 |
| β_2_ | 13.9214 | 13.3012 – 14.3279 |
| β_3_ | 0.3936 | 0.0065 – 1.0261 |
| $c_{1}$ | 0.0475 | 0.0038 – 0.1017 |
| E(0) | 0.0784 | 0.0046 – 0.1457 |
| A(0) | 7.6705 | 0.1492 – 23.4508 |
| I(0) | 0.6655 | 0.5707 – 0.7727 |
| α_1_ | 123.0122 | 3.461 – 258.6575 |
| α_2_ | 20.3504 | 11.9468 – 28.0994 |
|  |  |  |

S11 Table: Estimated parameters for the Model (B) with non-monotone incidence for the Riyadh province
