# Supplementary material for: A realistic two-strain model for MERS-CoV infection uncovers the high risk for epidemic propagation
Source: PLoS Negl Trop Dis. 2020 Feb 14;14(2):e0008065. doi: 10.1371/journal.pntd.0008065 (PMC7046297; doi:10.1371/journal.pntd.0008065)
Supplement: S12 Table — (DOCX) [file pntd.0008065.s012.docx]

| Parameters | Mean | 95% CI |
| --- | --- | --- |
| β_1_ | 19.4097 | 2.203 – 89.203 |
| $\rho$ | 0.6826 | 0.0745 – 0.9348 |
| β_2_ | 18.9631 | 0.0564 – 92.0066 |
| β_3_ | 4.4219 | 0.0862 – 12.4175 |
| $c_{1}$ | 0.2524 | 0.0624 – 0.3388 |
| E(0) | 1.8521 | 0.103 – 8.3664 |
| A(0) | 13.2865 | 0.8712 – 19.7526 |
| I(0) | 0.5376 | 0.057 – 0.9449 |
| α_1_ | 123.01 | 33.999 – 450.2641 |
| α_2_ | 276.1297 | 47.8241 – 470.9259 |
|  |  |  |

S12 Table: Estimated parameters for the Model (B) with saturated incidence for the Riyadh province
