# Supplementary material for: A realistic two-strain model for MERS-CoV infection uncovers the high risk for epidemic propagation
Source: PLoS Negl Trop Dis. 2020 Feb 14;14(2):e0008065. doi: 10.1371/journal.pntd.0008065 (PMC7046297; doi:10.1371/journal.pntd.0008065)
Supplement: S13 Table — (DOCX) [file pntd.0008065.s013.docx]

| Parameters | Mean | 95% CI |
| --- | --- | --- |
| β_1_ | 0.0229 | 0.0005 – 0.0834 |
| $\rho$ | 0.487 | 0.0244 – 0.9707 |
| β_2_ | 1.6274 | 1.5986 – 1.6722 |
| β_3_ | 0.1402 | 0.0046 – 0.4249 |
| $c_{1}$ | 0.0063 | 0.0002 – 0.0261 |
| E(0) | 13.2671 | 7.972 – 19.3387 |
| A(0) | 13.6753 | 0.5399 – 28.9644 |
| I(0) | 10.3693 | 8.2525 – 12.6119 |

S13 Table: Estimated parameters for the Model (B) with bilinear incidence for the Macca province
