# Supplementary material for: A realistic two-strain model for MERS-CoV infection uncovers the high risk for epidemic propagation
Source: PLoS Negl Trop Dis. 2020 Feb 14;14(2):e0008065. doi: 10.1371/journal.pntd.0008065 (PMC7046297; doi:10.1371/journal.pntd.0008065)
Supplement: S14 Table — (DOCX) [file pntd.0008065.s014.docx]

| Parameters | Mean | 95% CI |
| --- | --- | --- |
| β_1_ | 9.5006 | 0.6194 – 19.5597 |
| $\rho$ | 0.4875 | 0.0246 – 0.9731 |
| β_2_ | 10.2642 | 0.6376 – 19.7995 |
| β_3_ | 0.7823 | 0.0312 – 2.1551 |
| $c_{1}$ | 0.2340 | 0.0104 – 0.8723 |
| E(0) | 0.0257 | 0.0012 – 0.1 |
| A(0) | 1.6963 | 0.072 – 6.2675 |
| I(0) | 10.5456 | 9.1633 – 11.9072 |
| α_1_ | 269.8349 | 25.4294 – 490.1929 |
| α_2_ | 261.1533 | 25.4768 – 484.3788 |

S14 Table: Estimated parameters for the Model (B) with non-monotone incidence for the Macca province
