# Supplementary material for: A realistic two-strain model for MERS-CoV infection uncovers the high risk for epidemic propagation
Source: PLoS Negl Trop Dis. 2020 Feb 14;14(2):e0008065. doi: 10.1371/journal.pntd.0008065 (PMC7046297; doi:10.1371/journal.pntd.0008065)
Supplement: S15 Table — (DOCX) [file pntd.0008065.s015.docx]

| Parameters | Mean | 95% CI |
| --- | --- | --- |
| β_1_ | 13.8081 | 13.3566 – 14.3049 |
| $\rho$ | 0.7374 | 0.1912 – 0.8848 |
| β_2_ | 5.8951 | 4.9271 – 6.9907 |
| β_3_ | 0.4623 | 0.0428 – 0.8723 |
| $c_{1}$ | 0.0899 | 0.0041 – 0.4455 |
| E(0) | 0.1357 | 0.0067 – 0.6572 |
| A(0) | 0.0051 | 0.0002 – 0.0612 |
| I(0) | 10.6661 | 10.596 – 10.8292 |
| α_1_ | 75.8164 | 73.9574 – 76.5603 |
| α_2_ | 469.3787 | 468.8116 – 469.6643 |

S15 Table: Estimated parameters for the Model (B) with saturated incidence for the Macca province
