# Supplementary material for: A realistic two-strain model for MERS-CoV infection uncovers the high risk for epidemic propagation
Source: PLoS Negl Trop Dis. 2020 Feb 14;14(2):e0008065. doi: 10.1371/journal.pntd.0008065 (PMC7046297; doi:10.1371/journal.pntd.0008065)
Supplement: S16 Table — (DOCX) [file pntd.0008065.s016.docx]

| Parameters | Mean | 95% CI |
| --- | --- | --- |
| β_1_ | 0.0322 | 0.0013 – 0.08698 |
| $\rho$ | 5.38E-8 | 2.24E-9 – 1.82E-7 |
| β_2_ | 1.554 | 1.5358 – 1.583 |
| β_3_ | 3.67E-6 | 1.024E-8 – 2.367E-5 |
| $c_{1}$ | 3.02E-6 | 9.65E-8 – 1.8787E-5 |
| E(0) | 1.06E-6 | 5.89E-9 – 6.313E-6 |
| A(0) | 0.1696 | 0.00082 – 1.1365 |
| I(0) | 1.117 | 0.7 – 1.5464 |

S16 Table: Estimated parameters for the Model (B) with bilinear incidence for the Madina province
