# Supplementary material for: A realistic two-strain model for MERS-CoV infection uncovers the high risk for epidemic propagation
Source: PLoS Negl Trop Dis. 2020 Feb 14;14(2):e0008065. doi: 10.1371/journal.pntd.0008065 (PMC7046297; doi:10.1371/journal.pntd.0008065)
Supplement: S17 Table — (DOCX) [file pntd.0008065.s017.docx]

| Parameters | Mean | 95% CI |
| --- | --- | --- |
| β_1_ | 9.5787 | 0.3966 – 23.0179 |
| $\rho$ | 0.4471 | 0.01786 – 0.9647 |
| β_2_ | 12.5516 | 0.5035 – 23.26 |
| β_3_ | 0.0098 | 0.00037 – 0.06418 |
| $c_{1}$ | 0.05675 | 0.0016 – 0.1493 |
| E(0) | 0.02438 | 0.00046 – 0.1114 |
| A(0) | 16.072 | 0.9716 – 29.425 |
| I(0) | 1.4178 | 1.1356 – 1.7055 |
| α_1_ | 286.4767 | 40.8017 – 490.98 |
| α_2_ | 301.9506 | 73.3037 – 490.97 |

S17 Table: Estimated parameters for the Model (B) with non-monotone incidence for the Madina province
