# Supplementary material for: A realistic two-strain model for MERS-CoV infection uncovers the high risk for epidemic propagation
Source: PLoS Negl Trop Dis. 2020 Feb 14;14(2):e0008065. doi: 10.1371/journal.pntd.0008065 (PMC7046297; doi:10.1371/journal.pntd.0008065)
Supplement: S18 Table — (DOCX) [file pntd.0008065.s018.docx]

| Parameters | Mean | 95% CI |
| --- | --- | --- |
| β_1_ | 9.93 | 0.3474 – 21.55 |
| $\rho$ | 0.5125 | 0.0188 – 0.9783 |
| β_2_ | 11.8488 | 0.546 – 23.9459 |
| β_3_ | 0.1599 | 0.0074 – 0.4772 |
| $c_{1}$ | 0.04944 | 0.0011 – 0.1564 |
| E(0) | 0.15 | 0.0036 – 0.5104 |
| A(0) | 11.52 | 0.4108 – 29.257 |
| I(0) | 1.4188 | 1.1296 – 1.7286 |
| α_1_ | 435.0682 | 291.33 – 498.57 |
| α_2_ | 421.0263 | 147.83 – 499.62 |

S18 Table: Estimated parameters for the Model (B) with saturated incidence for the Madina province
