# Supplementary material for: A realistic two-strain model for MERS-CoV infection uncovers the high risk for epidemic propagation
Source: PLoS Negl Trop Dis. 2020 Feb 14;14(2):e0008065. doi: 10.1371/journal.pntd.0008065 (PMC7046297; doi:10.1371/journal.pntd.0008065)
Supplement: S19 Table — Model with smallest AIC and BIC value are given in bold. (DOCX) [file pntd.0008065.s019.docx]

| **Two strain** | **AIC (Weight =** $\mathbf{W}_{\mathbf{i}}$**)** | | | **BIC** | | |
| --- | --- | --- | --- | --- | --- | --- |
|  | Model 1 | Model 2 | Model 3 | Model 1 | Model 2 | Model 3 |
| Macca | **1885.72 (1)** | 2250.65  (0) | 2238.13  (0) | **1927.02** | 2297.46 | 2284.94 |
| Madina | **112.81**  **(1)** | 119.52  (0) | 121.26  (0) | **153.85** | 166.04 | 167.77 |
| Riyadh | 7633.14  (0) | **5854.53**  **(1)** | 6041.96  (0) | 7679.17 | **5906.7** | 6094.13 |
| **Single strain** | | | | | | |
| Macca | 4603.14  (0) | 2513.59  (0) | **2369.36**  **(1)** | 4625.16 | 2541.13 | **2396.9** |
| Madina | 249.93  (0) | **105.87**  **(1)** | 106.52  (0) | 271.82 | **133.24** | 133.88 |
| Riyadh | 6948.23  (0) | **5622.47**  **(1)** | 5961.21  (0) | 6972.78 | **5653.16** | 5991.9 |

S19 Table: Multi-model inference quantities (AIC and BIC) for three two-strain and single- strain models. Model with smallest AIC and BIC value are given in bold. For two strain models, Model -1 represents Model-(A) with bilinear incidence function. Model -2 represents Model-(A) with non-monotone incidence and Model -3 represents Model -(A) with saturated incidence. For single strain models, Model -1 represents Model-(B) with bilinear incidence function. Model -2 represents Model-(B) with non-monotone incidence and Model -3 represents Model -(B) with saturated incidence.
