# Supplementary material for: A realistic two-strain model for MERS-CoV infection uncovers the high risk for epidemic propagation
Source: PLoS Negl Trop Dis. 2020 Feb 14;14(2):e0008065. doi: 10.1371/journal.pntd.0008065 (PMC7046297; doi:10.1371/journal.pntd.0008065)
Supplement: S20 Table — The data is given in the format (Mean [95%CI]). (DOCX) [file pntd.0008065.s020.docx]

| **Province** | **Mean (R_0_)** | **95% CI** | **Mean (R_H_)** | **95% CI** | **Mean (R_C_)** | **95% CI** |
| --- | --- | --- | --- | --- | --- | --- |
| **Model 1** | | | | | | |
| Riyadh | 24.4308 | 7.6972 - 41.8678 | 0.4145 | 0.0279 - 1.1364 | 24.1896 | 7.6836 - 41.7325 |
| Macca | 1.9886 | 1.7524 - 2.1733 | 2.924e-05 | 1.48E-06 - 1.03E-05 | 1.9886 | 1.7524 - 2.1733 |
| Madina | 2.1093 | 1.8752 - 2.4584 | 1.08E-04 | 3.84E-06 - 3.52E-04 | 2.1092 | 1.8748 - 2.4584 |
| **Model 2** | | | | | | |
| Riyadh | 2.1870 | 1.3947- 3.2136 | 1.8052 | 0.1747 - 3.196 | 0.5482 | 0.0128 - 1.6255 |
| Macca | 5.4854 | 2.4851- 9.1844 | 0.0022 | 5.06E-5 - 0.0092 | 5.4838 | 2.4832 - 9.1806 |
| Madina | 5.4227 | 2.4399 - 9.6990 | 4.7397 | 0.3955 - 9.5913 | 1.1091 | 0.0469 - 2.5640 |

S20 Table: Estimated values of the Basic reproduction number (R_0_), the Hospital reproduction number (R_H_), and the Community reproduction number (R_C_), for the three provinces of Saudi Arabia for the two strain Model-1 and Model-2 (Equation (A) with bilinear incidence and non-monotone incidence). The data is given in the format (Mean [95%CI]).
