# Supplementary material for: A realistic two-strain model for MERS-CoV infection uncovers the high risk for epidemic propagation
Source: PLoS Negl Trop Dis. 2020 Feb 14;14(2):e0008065. doi: 10.1371/journal.pntd.0008065 (PMC7046297; doi:10.1371/journal.pntd.0008065)
Supplement: S21 Table — The data is given in the format (Mean [95%CI]). (DOCX) [file pntd.0008065.s021.docx]

| **Province** | **Mean (R_0_)** | | **95% CI** | **Mean (R_H_)** | **95% CI** | **Mean (R_C_)** | **95% CI** |
| --- | --- | --- | --- | --- | --- | --- | --- |
| **Model 1** | | | | | | | |
| Riyadh | 0.0377 | 0.0292 - 0.0430 | | 0.0065 | 0.0041 - 0.0078 | 0.0313 | 0.0247 - 0.0352 |
| Macca | 0.4959 | 0.4829 - 0.5204 | | 0.4899 | 0.4813 - 0.5034 | 0.0059 | 1.34E-4 - 0.0211 |
| Madina | 0.7690 | 0.7569 - 0.7899 | | 0.7650 | 0.7560 - 0.7793 | 0.0040 | 1.63E-4 - 0.0107 |
| **Model 2** | | | | | | | |
| Riyadh | 3.4018 | 3.2492 - 3.5007 | | 3.3989 | 3.2474 - 3.4981 | 0.0029 | 2.34E-4 - 0.0074 |
| Macca | 5.7474 | 3.2897 - 7.9113 | | 3.09 | 0.192 - 5.9606 | 2.6574 | 0.1383 - 7.0870 |
| Madina | 8.666 | 4.1889 - 11.9697 | | 6.1789 | 0.2478 - 11.4537 | 2.4871 | 0.0948 - 7.5064 |
| **Model 3** | | | | | | | |
| Riyadh | 10.5064 | 2.7915 - 43.9771 | | 4.6298 | 0.0138 - 22.4632 | 5.8766 | 0.7310 - 29.8462 |
| Macca | 6.7265 | 4.1782 - 7.4253 | | 1.7747 | 1.4833 - 2.1045 | 4.9518 | 2.5031 - 5.6222 |
| Madina | 8.7318 | 4.2287 - 12.3109 | | 5.8330 | 0.2688 - 11.7881 | 2.8989 | 0.0902 - 7.7587 |

S21 Table: Comparison of estimated values of the Basic reproduction number (R_0_), the Hospital reproduction number (R_H_), and the Community reproduction number (R_C_), for the three provinces of Saudi Arabia for the single strain Model-1, Model-2 and Model-3 (Equation (B) with bilinear, non-monotone and saturated incidence). The data is given in the format (Mean [95%CI]).
