# Supplementary material for: A realistic two-strain model for MERS-CoV infection uncovers the high risk for epidemic propagation
Source: PLoS Negl Trop Dis. 2020 Feb 14;14(2):e0008065. doi: 10.1371/journal.pntd.0008065 (PMC7046297; doi:10.1371/journal.pntd.0008065)
Supplement: S22 Table — Model -1 represents Model-(A) with bilinear incidence function. Model -2 represents Model-(A) with non-monotone incidence and Model -3 represents Model -(A) with saturated incidence. (DOCX) [file pntd.0008065.s022.docx]

|  | **Peak week* (weeks)** | **Peak incidence* (cases)** | **Total incidence (cases)** |
| --- | --- | --- | --- |
| **Observed values (Data)** | **51** | **4** | **20** |
| **Macca** |  |  |  |
| **Model 1** | 11.08 [39.9] | 6.70 [7.08] | 60.92 [63.42] |
| **Model 2** | 23.61 [27.5] | 17.41 [14.16] | 210.09 [191.11] |
| **Model 3** | 27.13 [23.9] | 18.23 [14.53] | 179.04 [159.04] |
| **Observed values (Data)** | **11** | **5** | **10** |
| **Madina** |  |  |  |
| **Model 1** | 28.26 [18.8] | 5.62 [3.25] | 76.59 [64.98] |
| **Model 2** | 21.59 [15.6] | 4.96 [2.62] | 37.40 [34.21] |
| **Model 3** | 17.93 [14.5] | 1.39 [3.6] | 10.14 [9.33] |

S22 Table: Average predictions [Simple average of Mean Absolute Errors (MAE)] obtained over all the prediction weeks using Model -(A) with different incidence functions. Model -1 represents Model-(A) with bilinear incidence function. Model -2 represents Model-(A) with non-monotone incidence and Model -3 represents Model -(A) with saturated incidence. * For peak week and peak incidence the simple average of MAE is provided up-to the peak of the prediction season.
