# Supplementary material for: A realistic two-strain model for MERS-CoV infection uncovers the high risk for epidemic propagation
Source: PLoS Negl Trop Dis. 2020 Feb 14;14(2):e0008065. doi: 10.1371/journal.pntd.0008065 (PMC7046297; doi:10.1371/journal.pntd.0008065)
Supplement: S23 Table — Model -1 represents Model-(B) with bilinear incidence function. Model -2 represents Model-(B) with non-monotone incidence and Model-3 represents Model-(B) with saturated incidence. (DOCX) [file pntd.0008065.s023.docx]

|  | **Peak week* (weeks)** | **Peak incidence* (cases)** | **Total incidence (cases)** |
| --- | --- | --- | --- |
| **Observed values (Data)** | **11** | **45** | **230** |
| **Riyadh** |  |  |  |
| **Model 1** | 29.58 [20.5] | 19.04 [25.96] | 299.85 [86.4] |
| **Model 2** | 19.31 [19.9] | 7.29 [37.71] | 215.18 [55.71] |
| **Model 3** | 30.58 [19.7] | 20.04 [24.95] | 233.88 [70.17] |
| **Observed values (Data)** | **51** | **4** | **20** |
| **Macca** |  |  |  |
| **Model 1** | 26.11 [24.9] | 20.65 [16.65] | 234.85 [214.85] |
| **Model 2** | 25.20 [26.1] | 7.85 [6.43] | 95.88 [76.95] |
| **Model 3** | 24.22 [19.7] | 3.97 [5.27] | 59.75 [49.76] |
| **Observed values (Data)** | **11** | **5** | **10** |
| **Madina** |  |  |  |
| **Model 1** | 29.05 [18.3] | 4.48 [1.28] | 37.38 [27.38] |
| **Model 2** | 18.03 [16.2] | 1.23 [3.79] | 15.01 [6.68] |
| **Model 3** | 18.08 [16.7] | 1.74 [3.33] | 11.45 [5.87] |

S23 Table: Average predictions [Simple average of Mean Absolute Errors (MAE)] obtained over all the prediction weeks using a single-strain Model - (B) with different incidence functions. Model -1 represents Model-(B) with bilinear incidence function. Model -2 represents Model-(B) with non-monotone incidence and Model-3 represents Model-(B) with saturated incidence. * For peak week and peak incidence the simple average of MAE is provided up-to the peak of the prediction season.
