# Supplementary material for: A realistic two-strain model for MERS-CoV infection uncovers the high risk for epidemic propagation
Source: PLoS Negl Trop Dis. 2020 Feb 14;14(2):e0008065. doi: 10.1371/journal.pntd.0008065 (PMC7046297; doi:10.1371/journal.pntd.0008065)
Supplement: S24 Table — (DOCX) [file pntd.0008065.s024.docx]

| Parameters | Mean | 95% CI |
| --- | --- | --- |
| β_1_ | 72.6195 | 58.8197 — 89.2830 |
| $\theta$ | 0.0038 | 4.6552e-04 – 0.0088 |
| $\rho$ | 0.6051 | 0.2006 – 0.9077 |
| β_2_ | 6.5032 | 4.8437 – 8.2993 |
| β_3_ | 0.8271 | 0.0366 – 2.1517 |
| $p_{1}$ | 4.5578e-06 | 5.2455e-07 – 8.7950e-06 |
| $p_{2}$ | 7.0081e-05 | 6.0457e-06 – 1.5771e-04 |
| $c_{1}$ | 0.0154 | 0.0068 – 0.0291 |
| $c_{2}$ | 0.2137 | 0.0294 – 0.5226 |
| E_1_(0) | 5.0129e-04 | 2.5469e-05 – 0.0010 |
| E_2_(0) | 4.1220 | 2.5432 – 4.9835 |
| A_1_(0) | 2.7494e-04 | 9.7890e-05– 4.7241e-04 |
| A_2_(0) | 0.0350 | 0.0037 – 0.0809 |
| I_1_(0) | 4.8919e-06 | 3.5153e-07– 1.5250e-05 |
| I_2_(0) | 0.0752 | 0.0373 – 0.1328 |
| I_3_(0) | 5.3028e-05 | 2.8158e-06 – 1.0900e-04 |
| Η | 3.6829 | 1.9835 – 5.3780 |
| Φ | 0.3230 | 0.0894 – 0.6764 |

S24 Table: Estimated parameters for Model-(A1) for Riyadh
