# Supplementary material for: A realistic two-strain model for MERS-CoV infection uncovers the high risk for epidemic propagation
Source: PLoS Negl Trop Dis. 2020 Feb 14;14(2):e0008065. doi: 10.1371/journal.pntd.0008065 (PMC7046297; doi:10.1371/journal.pntd.0008065)
Supplement: S25 Table — (DOCX) [file pntd.0008065.s025.docx]

| Parameters | Mean | 95% CI |
| --- | --- | --- |
| β_1_ | 14.6897 | 14.1412 — 15.2507 |
| $\theta$ | 9.3379e-08 | 3.5816e-09 – 1.9775e-07 |
| $\rho$ | 0.2104 | 0.0343 – 0.5000 |
| β_2_ | 0.0104 | 3.7671e-04 – 0.0369 |
| β_3_ | 0.5093 | 0.1377 – 0.8097 |
| $p_{1}$ | 0.1615 | 0.0245 – 0.3791 |
| $p_{2}$ | 0.0034 | 1.1080e-04 – 0.0202 |
| $c_{1}$ | 0.0585 | 0.0286 – 0.1589 |
| $c_{2}$ | 3.8048e-04 | 3.5136e-05 – 8.6003e-04 |
| E_1_(0) | 0.0016 | 1.1805e-04 – 0.0028 |
| E_2_(0) | 4.5899e-04 | 2.1876e-05– 9.0599e-04 |
| A_1_(0) | 8.3381e-04 | 1.3638e-04 – 0.0019 |
| A_2_(0) | 3.6675 | 0.8504 – 7.1788 |
| I_1_(0) | 0.0019 | 3.9468e-04 – 0.0034 |
| I_2_(0) | 11.4552 | 10.9833 – 11.8047 |
| I_3_(0) | 8.5004e-07 | 3.6579e-08 – 2.2481e-06 |
| Η | 3.2655 | 1.8846 – 5.5201 |
| Φ | 0.5433 | 0.2262 – 0.7894 |

S25 Table: Estimated parameters for Model-(A1) for Macca
