# Supplementary material for: A realistic two-strain model for MERS-CoV infection uncovers the high risk for epidemic propagation
Source: PLoS Negl Trop Dis. 2020 Feb 14;14(2):e0008065. doi: 10.1371/journal.pntd.0008065 (PMC7046297; doi:10.1371/journal.pntd.0008065)
Supplement: S26 Table — (DOCX) [file pntd.0008065.s026.docx]

| Parameters | Mean | 95% CI |
| --- | --- | --- |
| β_1_ | 15.6019 | 14.6866 — 16.7483 |
| $\theta$ | 2.6504e-05 | 6.7550e-07 – 9.6350e-05 |
| $\rho$ | 9.2251e-08 | 6.6290e-09 – 2.4392e-07 |
| β_2_ | 2.6136e-04 | 1.0807e-05 – 6.8756e-04 |
| β_3_ | 0.0628 | 0.0137 – 0.1168 |
| $p_{1}$ | 0.6349 | 0.0441 – 0.9824 |
| $p_{2}$ | 0.0277 | 7.9789e-04 – 0.0756 |
| $c_{1}$ | 3.8811e-04 | 1.8284e-05 – 9.7037e-04 |
| $c_{2}$ | 4.8221e-04 | 3.3185e-05 – 9.9440e-04 |
| E_1_(0) | 0.0038 | 3.9068e-04 – 0.0060 |
| E_2_(0) | 0.0024 | 5.9940e-05 – 0.0088 |
| A_1_(0) | 0.0045 | 4.6597e-04 – 0.0085 |
| A_2_(0) | 17.6982 | 2.0159 – 29.4074 |
| I_1_(0) | 1.2796e-04 | 4.6183e-06 – 4.2137e-04 |
| I_2_(0) | 1.5718 | 1.4574 – 1.6721 |
| I_3_(0) | 2.8037e-04 | 1.6595e-05 – 5.7976e-04 |
| Η | 1.0569 | 1.0022 – 1.1574 |
| Φ | 0.6986 | 0.0587 – 0.8764 |

S26 Table: Estimated parameters for Model-(A1) for Madina
