# Supplementary material for: A realistic two-strain model for MERS-CoV infection uncovers the high risk for epidemic propagation
Source: PLoS Negl Trop Dis. 2020 Feb 14;14(2):e0008065. doi: 10.1371/journal.pntd.0008065 (PMC7046297; doi:10.1371/journal.pntd.0008065)
Supplement: S27 Table — (DOCX) [file pntd.0008065.s027.docx]

| Parameters | Mean | 95% CI |
| --- | --- | --- |
| β_1_ | 0.0031 | 2.5786e-04 – 0.0084 |
| $\rho$ | 0.5228 | 0.4368 – 0.5603 |
| β_2_ | 0.8497 | 0.7054 – 0.9137 |
| β_3_ | 3.7176 | 3.5838 – 4.0235 |
| $c_{1}$ | 4.9437e-04 | 2.4276e-04 – 7.9356e-04 |
| E(0) | 0.0760 | 0.0111 – 0.1244 |
| A(0) | 13.0016 | 11.3854 – 13.7130 |
| I(0) | 3.6389 | 3.5729 – 3.8225 |
| I_3_(0) | 1.1885 | 1.0772 – 1.2326 |
| Η | 1.9563 | 1.8774 – 2.0315 |
| Φ | 0.7538 | 0.7440 – 0.7804 |

S27 Table: Estimated parameters for the Model (B1) for Riyadh
