# Supplementary material for: A realistic two-strain model for MERS-CoV infection uncovers the high risk for epidemic propagation
Source: PLoS Negl Trop Dis. 2020 Feb 14;14(2):e0008065. doi: 10.1371/journal.pntd.0008065 (PMC7046297; doi:10.1371/journal.pntd.0008065)
Supplement: S28 Table — (DOCX) [file pntd.0008065.s028.docx]

| Parameters | Mean | 95% CI |
| --- | --- | --- |
| β_1_ | 0.0975 | 0.0018 – 0.2806 |
| $\rho$ | 0.0493 | 0.0017 – 0.2208 |
| β_2_ | 0.0443 | 0.0011 – 0.1836 |
| β_3_ | 10.3640 | 9.8946 – 10.6390 |
| $c_{1}$ | 0.0209 | 0.0011 – 0.0551 |
| E(0) | 0.4738 | 0.0063 – 2.7184 |
| A(0) | 1.5030 | 0.0500 – 7.4463 |
| I(0) | 0.1457 | 0.0050 – 0.5919 |
| I_3_(0) | 0.0844 | 0.0015 – 0.3329 |
| Η | 1.8073 | 1.0156 – 3.5746 |
| Φ | 0.6909 | 0.1959 – 0.9792 |

S28 Table: Estimated parameters for the Model (B1) for Macca
