# Supplementary material for: A realistic two-strain model for MERS-CoV infection uncovers the high risk for epidemic propagation
Source: PLoS Negl Trop Dis. 2020 Feb 14;14(2):e0008065. doi: 10.1371/journal.pntd.0008065 (PMC7046297; doi:10.1371/journal.pntd.0008065)
Supplement: S29 Table — (DOCX) [file pntd.0008065.s029.docx]

| Parameters | Mean | 95% CI |
| --- | --- | --- |
| β_1_ | 0.0432 | 0.0013 – 0.1278 |
| $\rho$ | 0.0105 | 3.4607e-04 – 0.0337 |
| β_2_ | 0.0729 | 0.0063 – 0.1504 |
| β_3_ | 0.0135 | 3.3896e-04 – 0.0495 |
| $c_{1}$ | 1.2250e-05 | 9.9005e-07 – 2.4433e-05 |
| E(0) | 0.1117 | 0.0047 – 0.3726 |
| A(0) | 0.1575 | 0.0102 – 0.4186 |
| I(0) | 1.2073 | 0.2617 – 1.8136 |
| I_3_(0) | 0.7318 | 0.1198 – 1.6445 |
| Η | 1.7413 | 1.0689 – 2.6563 |
| Φ | 0.5575 | 0.0497 – 0.9641 |

S29 Table: Estimated parameters for the Model (B1) for Madina
