# Supplementary material for: A realistic two-strain model for MERS-CoV infection uncovers the high risk for epidemic propagation
Source: PLoS Negl Trop Dis. 2020 Feb 14;14(2):e0008065. doi: 10.1371/journal.pntd.0008065 (PMC7046297; doi:10.1371/journal.pntd.0008065)
Supplement: S1 Fig — R0 is estimated over different forecast weeks (0, 4, 8, …, 48). (DOCX) [file pntd.0008065.s030.docx]

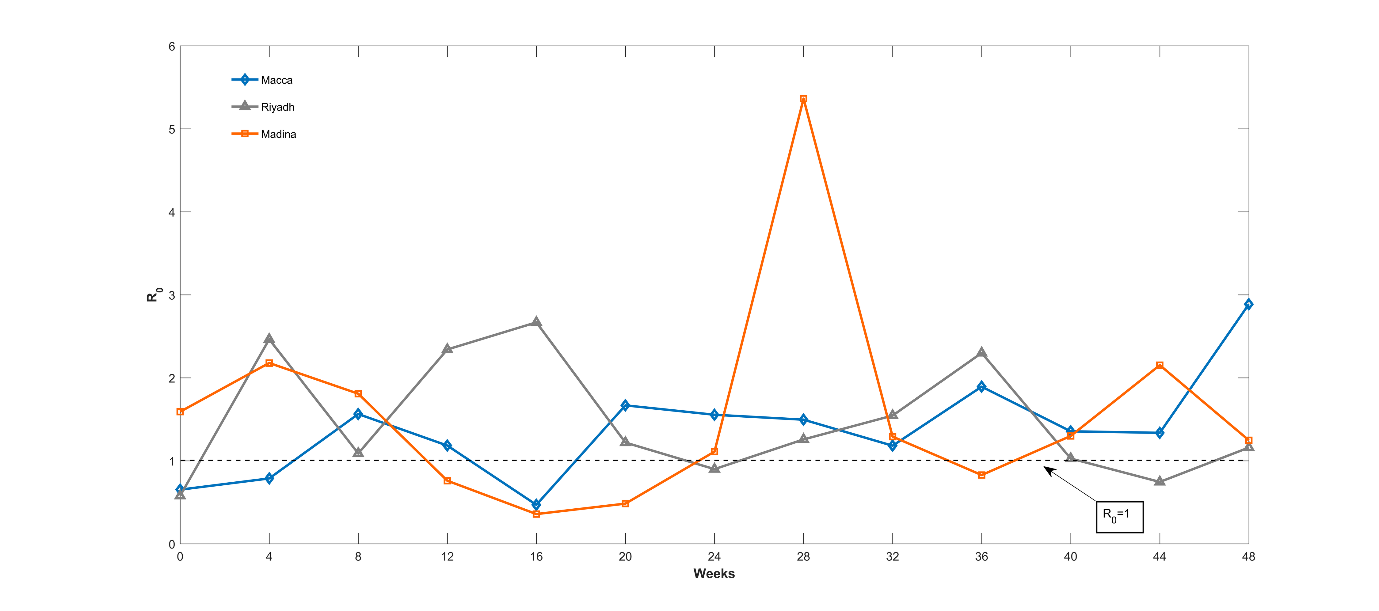


S1 Fig. Temporal Evolution of R_0_ using the best predicted 2-strain model (Saturated incidence) in three provinces; Riyadh, Macca and Madina. R_0_ is estimated over different forecast weeks (0, 4, 8,..., 48). Dotted line represents the threshold of the epidemic potential (R_0_ =1).
