# Supplementary material for: A realistic two-strain model for MERS-CoV infection uncovers the high risk for epidemic propagation
Source: PLoS Negl Trop Dis. 2020 Feb 14;14(2):e0008065. doi: 10.1371/journal.pntd.0008065 (PMC7046297; doi:10.1371/journal.pntd.0008065)
Supplement: S2 Fig — Dotted line represents the threshold of the epidemic potential (RH = 1). (DOCX) [file pntd.0008065.s031.docx]

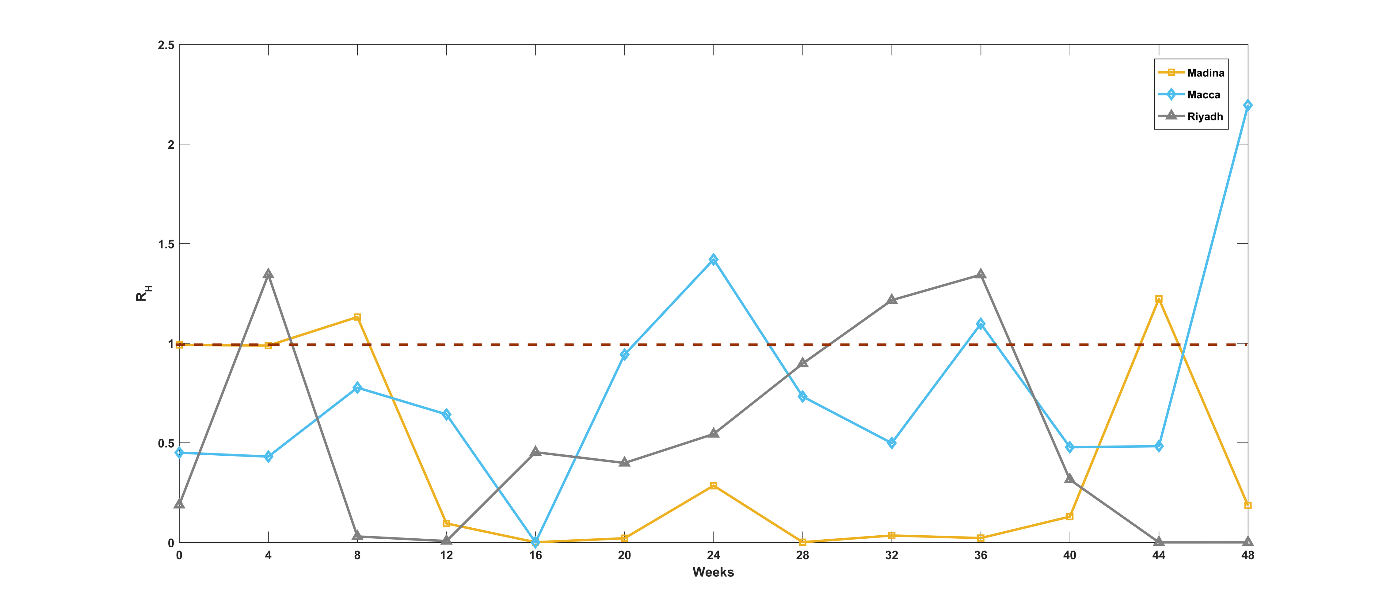


S2 Fig. Temporal Evolution of Hospital reproduction number (R_H_) using the best predicted 2-strain model (Saturated incidence) in three provinces; Riyadh, Macca and Madina. R_H_ is estimated over different forecast weeks (0, 4, 8,..., 48). Dotted line represents the threshold of the epidemic potential (R_H_ =1).
