# Supplementary material for: A realistic two-strain model for MERS-CoV infection uncovers the high risk for epidemic propagation
Source: PLoS Negl Trop Dis. 2020 Feb 14;14(2):e0008065. doi: 10.1371/journal.pntd.0008065 (PMC7046297; doi:10.1371/journal.pntd.0008065)
Supplement: S4 Fig — (DOCX) [file pntd.0008065.s033.docx]

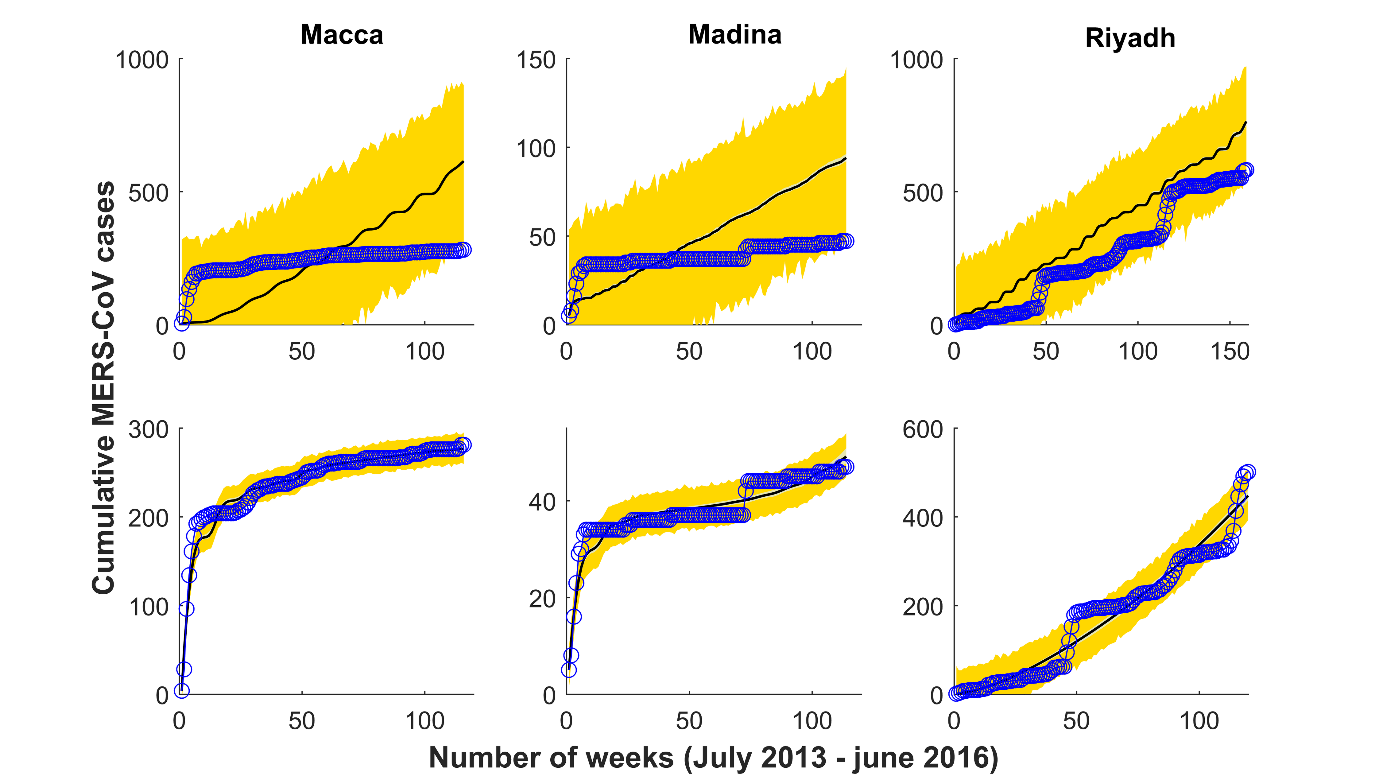


S4 Fig. Model simulations fitted to accumulated MERS-CoV clinical cases in Macca, Madina and Riyadh. Observed data points are shown in blue and the solid line depicts the model solutions. Two models fitted to cumulative MERS-CoV cases are: B1: single strain model with super-spreaders (three panels on the first row) and A1: two strain model with superspreaders (three panels on the second row).
